# Estimating the global impact of poor quality of care on maternal and neonatal outcomes in 81 low- and middle-income countries: A modeling study

**DOI:** 10.1371/journal.pmed.1002990

**Published:** 2019-12-18

**Authors:** Victoria B. Chou, Neff Walker, Mufaro Kanyangarara

**Affiliations:** Department of International Health, Institute for International Programs, Johns Hopkins Bloomberg School of Public Health, Baltimore, Maryland, United States of America; London School of Hygiene and Tropical Medicine, UNITED KINGDOM

## Abstract

**Background:**

In low-resource settings where disease burdens remain high and many health facilities lack essentials such as drugs or commodities, functional equipment, and trained personnel, poor quality of care often results and the impact can be profound. In this paper, we systematically quantify the potential gain of addressing quality of care globally using country-level data about antenatal, childbirth, and postnatal care interventions.

**Methods and findings:**

In this study, we created deterministic models to project health outcomes if quality of care was addressed in a representative sample of 81 low- and middle-income countries (LMICs). First, available data from health facility surveys (e.g., Service Provision Assessment [SPA] and Service Availability and Readiness Assessment [SARA]) conducted 2007–2016 were linked to household surveys (e.g., Demographic and Health Surveys [DHS] and Multiple Indicator Cluster Surveys [MICS]) to estimate baseline coverage for a core subset of 19 maternal and newborn health interventions. Next, models were constructed with the Lives Saved Tool (LiST) using country-specific baseline levels in countries with a linked dataset (*n* = 17) and sample medians applied as a proxy in countries without linked data. Lastly, these 2016 starting baseline levels were raised to reach targets in 2020 as endline based upon country-specific utilization (e.g., proportion of women who attended 4+ antenatal visits, percentage of births delivered in a health facility) from the latest DHS or MICS population-based reports. Our findings indicate that if high-quality health systems could effectively deliver this subset of evidence-based interventions to mothers and their newborns who are already seeking care, there would be an estimated 28% decrease in maternal deaths, 28% decrease in neonatal deaths, and 22% fewer stillbirths compared to a scenario without any change or improvement in quality of care. Totals of 86,000 (range, 77,800–92,400) maternal and 0.67 million (range, 0.59 million–0.75 million) neonatal lives could be saved, and 0.52 million (range, 0.48 million–0.55 million) stillbirths could be prevented across the 81 countries in the calendar year 2020 when adequate quality care is provided at current levels of utilization. Limitations include the paucity of data to individually assess quality of care for each intervention in all LMICs and the necessary assumption that quality of care being provided among the subset of countries with linked datasets is comparable or representative of LMICs overall.

**Conclusions:**

Our findings suggest that efforts to close the quality gap would still produce substantial benefits at current levels of access or utilization. With estimated mortality rate declines of 21%–32% on average, gains from this first step would be significant if quality was improved for selected antenatal, intrapartum, and postnatal interventions to benefit pregnant women and newborns seeking care. Interventions provided at or around the time of childbirth are most critical and accounted for 64% of the impact overall estimated in this quality improvement analysis.

## Abstract 2

Victoria Chou and colleagues estimate the implications of poor-quality care for maternal and child health outcomes in low- and middle-income countries.

## Author summary

**Why was this study done?:**

- In low- and middle-income countries, health systems struggle to provide high-quality medical care to pregnant women and their newborns in need.
- Delivery of key health interventions is critical to save lives and prevent morbidity and mortality in high-burden settings.
- The impact of extant or poor quality of care at a population level is poorly understood, and quantifying the benefits of improving quality among those already seeking or accessing care would be a critical first step for prioritization.

**What did the researchers do and find?:**

- To examine the global impact of improvement in the quality of care, we used a linking approach, which combines health facility and population-level survey data to estimate baseline for a subset of important evidence-based interventions.
- Intervention coverage trends were modeled in 81 countries by setting current levels of reported utilization as the final country-specific targets for 2020.
- If those seeking medical attention arrived at facilities reimagined with adequate resources and receive high-quality care, our systematic tally indicates that almost one quarter of the maternal deaths, neonatal deaths, and stillbirths would be preventable during the period 2016–2020 if the gaps in quality of care were eliminated.

**What do these findings mean?:**

- Countries and current health systems are far from ensuring that skilled providers with adequate supplies are providing timely and appropriate healthcare to existing populations in need. Our analysis of potential gains quantifies the consequences of these missed opportunities, ranging from the prenatal to postnatal periods.
- Bolstering the quality of care is an essential checkpoint because efforts to increase utilization will rely on the same health systems where vulnerable populations are presently accessing care.
- With greater attention focused on tracking country-level progress, more data will hopefully become available to effectively monitor changes in coverage for key maternal and neonatal interventions. As these parameters or inputs are better defined, modeling can contribute to the body of knowledge by offering an informed approach to examine quality of care gaps so that better strategies can be developed to improve health among mothers and their children seeking care in this context.

## Introduction

Achieving targets for the Sustainable Development Goals (SDGs) for 2030 will require not only rapid accelerated change but also robust linkages established across many sectors. The central importance of a high-quality, well-functioning, and resilient healthcare system to deliver proven interventions was championed over a decade ago by the World Health Organization (WHO) [1], and the urgent need for this strong foundation has become increasingly apparent as information about coverage of available interventions is systematically tracked [2] and trends across and within countries are documented [3]. Gaps are apparent, as health outcomes have not improved despite encouraging signs such as a greater proportion of births occurring in health facilities [4]. Simply knowing what should be done to reduce maternal [5] and neonatal mortality [6] and to prevent stillbirths [7] in low-resource settings is not sufficient when countries face the concomitant challenges of both providing those effective interventions at scale and maintaining a tolerable threshold for the quality of medical care being provided.

Health systems strengthening and the focus on an integrated approach coincides with increasing awareness that quality of care is a complex, multifactorial, but critical driver that can catalyze progress or mire potential success [8]. The Lancet Global Health Commission on High Quality Health Systems, which called for a better understanding of the dimension of quality for health systems in resource-limited settings [9], has contributed growing evidence that merely accessing or reaching the doorstep of a healthcare system does not equate to or ensure receipt of high-quality care [10,11].

WHO’s vision for pregnant women and newborns centers on provision of established life-saving interventions through high-quality and timely care to effectively reduce leading causes of mortality and morbidity worldwide [12]. Quality of care can be broadly conceptualized as structure including material resources or other structural inputs, processes or the activities undertaken by patient and/or provider, and outcomes defined as change(s) in resulting health status [13]. In low-resource settings, where fragmented health programs face shortfalls in drugs and supplies, functional equipment, and trained personnel, ensuring adequate quality of care within health systems is a key stepping stone if the ultimate benefits of achieving universal health coverage are to be successfully maximized [14].

**Fig 1:**
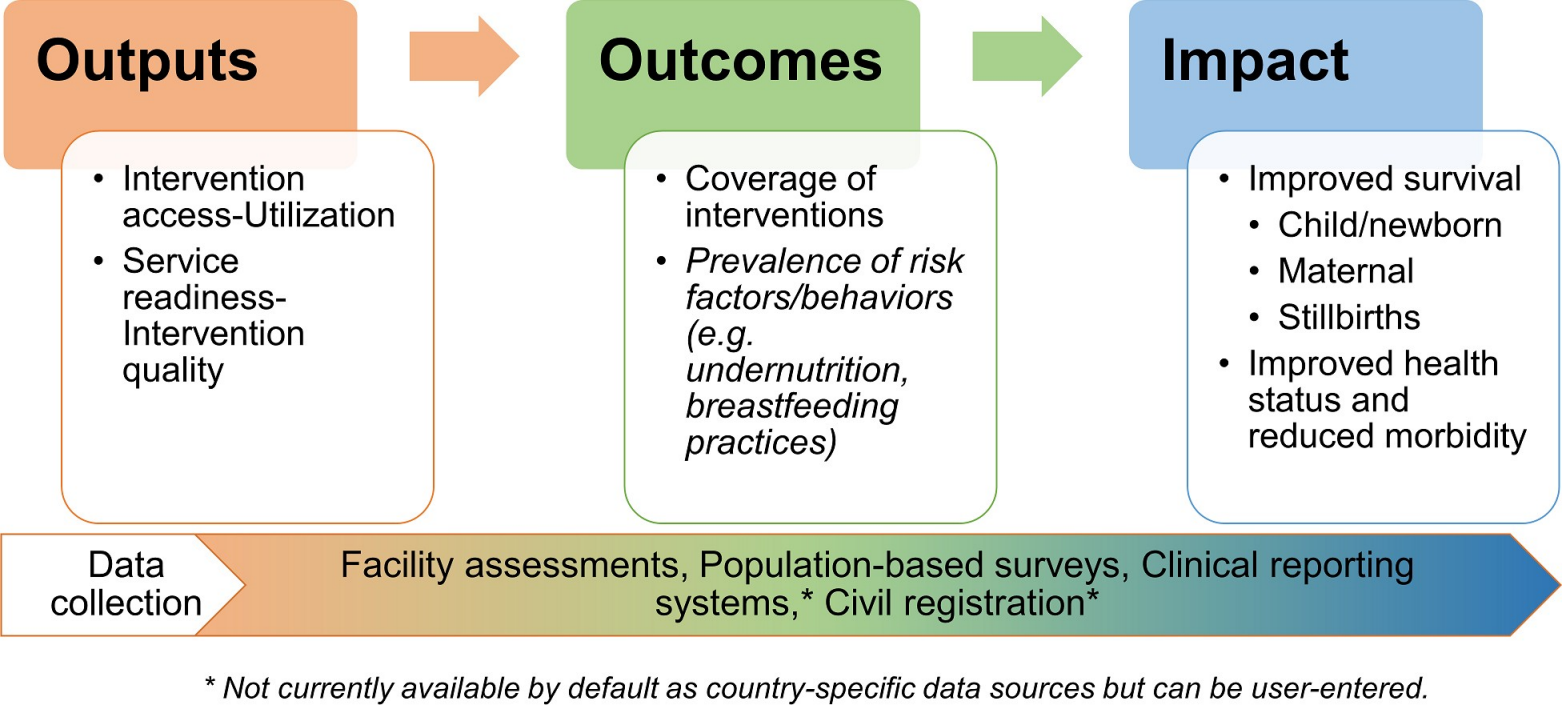
Framework for evaluation of health systems. The conceptual model of the LiST depicted with applicable data sources. LiST, Lives Saved Tool.

To this end, mathematical models have served as a valuable platform, because outcomes can be mapped into the future and measurable gains can be quantified for evaluation or strategic planning purposes [15]. Projecting the potential impact of interventions was first presented by the Bellagio Child Survival Group [16] and this framework was further developed and expanded into the subsequent Lives Saved Tool (LiST) model [17]. This evidence-based approach has traditionally focused on the “outcomes to impact” linkage (Fig 1), with LiST extrapolating the impact of scaling up coverage of key maternal, neonatal, and child health (MNCH) interventions with baseline levels typically derived from household surveys or country-level reporting.

The aim of this study is to estimate the global impact of poor quality of care in countries where disease burdens remain high and elevated morbidity and mortality persist. To achieve this aim, we conducted a multicountry analysis with a novel application combining elements from health facility assessments with utilization data to first examine intervention coverage. This insight is then used to estimate impact (i.e., improved survival) if points of contact made with the existing healthcare system during pregnancy and labor were no longer serving as empty placeholders. Our modeling approach explores the global consequences if the quality gap could be closed so that “effective coverage,” or the “fraction of potential health gain that is actually delivered to the population through the health system” [18], could be expanded during the period 2016–2020 to merely represent those already seeking care in each country.

## Methods

### Study design and sample

The LiST, a linear and deterministic model [17], was used to estimate the impact that improved quality of care could have for MNCH. The study protocol and analysis plan were not prespecified and were not available prior to this study. The RECORD statement is available in S1 RECORD Checklist as a checklist, but ethics approval was not required for this analysis. This modeling exercise focused on secondary data analysis of large, cross-sectional, publicly available, and nationally representative survey datasets, which do not require ethical review for analysis. The LiST model’s primary determinant is intervention coverage, which is holistically defined as, “the proportion of a population in need of a health intervention that actually receives it” [15], and increases in population-level intervention coverage are used to calculate impact, which is quantified as a reduction in the number of deaths or other adverse outcomes (e.g., preterm birth). The model is routinely revised to incorporate updated country-specific information about intervention coverage from household surveys (e.g., Demographic and Health Surveys [DHS], Multiple Indicator Cluster Survey [MICS]) and estimates of cause-specific intervention effectiveness [19]. Default data sources for standard model parameters are presented in S1 Table. LiST module from Version 5.67, released May 10, 2018, as part of the Spectrum suite, was used for this analysis.

Country-specific models for a sample of 81 low- and middle-income countries (LMICs) representing the majority of the global burden being tracked by Countdown to 2030 were created (see S2 Table), with most contextual indicators about population health status and intervention coverage drawn from national level surveys. Of the global burden, this subset of countries represents an estimated 89% of the neonatal deaths, 96% of the maternal deaths, and 87% of the stillbirths occurring worldwide [20,21].

### Baseline parameters

Using a linking approach, nationally representative data for the study period from 2007 to 2015 were collated from health facility assessments (i.e., Service Provision Assessment [SPA] and Service Availability and Readiness Assessment [SARA] surveys) and then combined with data from household surveys (e.g., DHS and MICS). Linked surveys were separated by an interval of ±2 years or less, and countries with linked datasets available (*n* = 17) are shown in Table 1.

**Table 1:** Linked datasets used to estimate baseline coverage of interventions.

Country	Health facility survey	Household survey	Antenatal care interventions	Childbirth care and postnatal care interventions
Bangladesh	SPA 2014	2014 DHS	YES	YES
Benin	SARA 2013	2011–2012 DHS	YES	YES
Burkina Faso	SARA 2012	2010 DHS	YES	YES
Democratic Republic of Congo	SARA 2014	2013–2014 DHS	YES	YES
Haiti	SPA 2013	2012 DHS	YES	YES
Kenya	SPA 2010	2008–2009 DHS	YES	-
Malawi	SPA 2013	2015–2016 DHS	YES	YES
Mauritania	SARA 2012	2011 MICS	-	YES
Namibia	SPA 2009	2006–2007 DHS	YES	-
Nepal	SPA 2015	2016 DHS	YES	YES
Rwanda	SPA 2007	2007–2008 DHS	YES	-
Senegal	SPA 2016	2016 DHS	YES	YES
Sierra Leone	SARA 2013	2013 DHS	YES	YES
Tanzania	SPA 2014	2015–2016 DHS	YES	YES
Togo	SARA 2012	2013–2014 DHS	YES	YES
Uganda	SARA 2012	2011 DHS	YES	YES
Zimbabwe	SARA 2014	2015 DHS	YES	YES

Abbreviations: DHS, Demographic and Health Surveys; MICS, Multiple Indicator Cluster Surveys; SARA, Service Availability and Readiness Assessment; SPA, Service Provision Assessment

A full description of the linking approach used to derive estimates of population-based intervention coverage has been published [22,23], but in summary, uniform indicator definitions were developed to specify a minimum set of structural inputs for particular antenatal, childbirth, or postnatal care interventions (*n* = 19). This subset of 19 life-saving maternal and neonatal health interventions was selected because the availability of key components (e.g., required staff and guidelines, equipment, medicines, and commodities) was assessed during SPA or SARA data collection and indicators could be constructed for readiness as a proxy for the quality of care. These interventions were drawn from the complete listing of 60+ interventions available in the module (see S1 Text documentation), which are organized across the continuum of care by timing of provision: from antenatal care, childbirth care, to postnatal preventive or curative care. One single intervention may impact one of more causes of death (e.g., neonatal sepsis and neonatal tetanus) or influence one or more outcomes (e.g., maternal death and stillbirths). One outcome may be impacted by more than one intervention, but the overall structure of the cause-specific mortality model precludes errors attributable to double counting.

Basic elements assumed to be required for adequate delivery of each selected health intervention are presented in Table 2. For example, the availability of a functional newborn bag and mask, staff trained in neonatal resuscitation, and Integrated Management of Pregnancy and Childbirth (IMPAC) guidelines are assumed to be the minimum required for the provision of neonatal resuscitation as a life-saving intervention. “Baseline” levels of coverage were then calculated by multiplying the availability of these essential components as a measure of readiness by the proportion of pregnant women seeking care or births occurring at this level within each health facility stratum. Accounting for both utilization and readiness with this approach provides an approximation of how high intervention coverage could theoretically be, given the prevailing restrictions of missing components or low levels of care-seeking. These analyses were conducted using STATA 14.2 (College Station, TX).

**Table 2:** Uniform indicator definitions applied for each intervention.

**Antenatal care (ANC)**	
**Intervention**	**Definition**
**Tetanus toxoid vaccination**	Observed at least one valid unexpired unit of tetanus toxoid vaccine
	At least one staff member trained in at least one aspect of ANC
	Reported availability of ANC guidelines
**Intermittent preventive treatment of malaria during pregnancy **	Observed at least one valid unexpired unit of sulphadoxine/pyrimethamine (SP)
	At least one staff member trained in at least one aspect of ANC
	Reported availability of ANC guidelines
**Syphilis detection and treatment **	Observed at least one valid syphilis test (RDT, RPR, or VDRL)
	Observed at least one valid unexpired unit of injectable penicillin (benzathine penicillin or procaine penicillin)
	At least one staff member trained in at least one aspect of ANC
	Reported availability of ANC guidelines
**Iron supplementation in pregnancy **	Observed at least one valid unexpired unit of iron or iron and folic acid tablets
	At least one staff member trained in at least one aspect of ANC
	Reported availability of ANC guidelines
**Hypertensive disorder case management **	Observed at least one valid dipstick for urine protein
	Observed at least one functioning blood pressure apparatus
	Observed at least one valid unexpired unit of amlodipine/nifedipine/methyldopa
	At least one staff member trained in at least one aspect of ANC
	Reported availability of ANC guidelines
**Diabetes case management **	Observed at least one glucometer AND glucometer test strips
	Observed at least one valid dipstick for urine glucose
	At least one staff member trained in at least one aspect of ANC
	Reported availability of ANC guidelines
**Malaria case management **	Observed at least one RDT kit; or smear with microscope, slides, and Wright Giemsa stain
	Observed at least one valid unexpired unit of Artemisinin-based Combination Therapy (ACT)
	At least one staff member trained in at least one aspect of ANC
	Reported availability of ANC guidelines
**MgSO4 management of preeclampsia **	Observed at least one valid unexpired unit of magnesium sulfate
	At least one staff member trained in at least one aspect of ANC
	Reported availability of ANC guidelines
**Childbirth (CB) care**	
**Intervention**	**Definition**
**Clean birth practices**	Observed availability of guidelines for IMPAC
	Observed availability of guidelines on standard precautions for infection prevention
	Observed availability of soap and running water or gloves or alcohol-based hand rub
	Observed availability and reported functionality of either a dry heat sterilizer or an autoclave
**Labor and delivery management**	Observed availability of blank partographs
	Observed availability of at least one delivery pack or all the following individual equipment: cord clamp, episiotomy scissors, scissors or blade to cut cord, suture material with needle, and needle holder.
	Observed availability of a delivery bed
	Observed availability and reported functionality of a spotlight source (or flashlight)
	Observed availability of guidelines IMPAC
**Neonatal resuscitation**	At least one staff member trained for neonatal resuscitation in past 2 years (SPA only)
	Observed availability and reported functionality of a newborn bag and mask
	Observed availability of guidelines for IMPAC
**Antibiotics for pPRoM**	Observed at least one valid unexpired unit of azithromycin
	Observed availability of guidelines for IMPAC
**Management of eclampsia with MgSO4**	Observed availability of at least one valid unit of injectable magnesium sulfate in service area or where routinely stocked
	Observed availability of guidelines for IMPAC
**Active management of the third stage of labor (AMTSL)**	At least one staff member trained in AMTSL in past 2 years (SPA only)
	Observed availability of at least one valid unit of injectable uterotonic (oxytocin or other) or oral misoprostol
	Observed availability of guidelines for IMPAC
**Induction of labor for pregnancies lasting 41+ weeks**	Observed availability of at least one valid unit of injectable uterotonic (oxytocin or other) or oral misoprostol
	Observed availability of guidelines for IMPAC
**Postnatal care**	
**Intervention**	**Definition**
**Hygienic cord care**	Observed availability of at least one valid unexpired unit of chlorhexidine (SPA only)
	Observed availability of guidelines for IMPAC
**Thermal care for case management of premature babies**	Reported routinely observing the drying/wrapping or skin-to-skin of newborns (SPA only)
	Observed availability of guidelines for IMPAC
**Kangaroo Mother Care for case management of premature babies**	At least one staff member trained in Kangaroo Mother Care in past 2 years (SPA only)
	Observed availability of guidelines for IMPAC
**Case management of neonatal sepsis/pneumonia with injectable antibiotics**	Observed availability of at least one valid unexpired unit of procaine benzylpenicillin or gentamicin and ceftriaxone
	Observed availability of guidelines for IMPAC

Abbreviations: IMPAC, Integrated Management of Pregnancy and Childbirth; pPRoM, preterm premature rupture of the membranes; RDT, rapid diagnostic test; RPR, rapid plasma reagin; SPA, Service Provision Assessment; VDRL, venereal disease research laboratory

### Scenarios for scaling up

Baseline levels for intervention coverage were calculated and applied in countries with a linked dataset, and the median for each intervention was calculated based upon this sample. For countries without a linked dataset, the sample median was applied to serve as a proxy for the starting level in 2016. The 25th and 75th percentile values were also calculated, and sensitivity analyses were conducted to examine these thresholds as lower and upper bounds, respectively. For all 81 countries included in the analysis, a country-specific national target or hypothetical “cap” was set for 2020 based upon reported access or utilization (see S1 Table for the most recent survey source). The proportion of women who attended four or more antenatal visits (ANC4+) according to the last population-based household survey was considered a standard measure of the percent of pregnant women who “should have” properly received evidence-based antenatal interventions during an antenatal visit. Similarly, the percentage of births delivered in a health facility (HFD) was considered the endpoint for modeling intervention scale-up as improvements in the quality of childbirth interventions based upon the conservative assumption that every individual who sought care “should have” received care of adequate quality during the intrapartum period. Annual trends starting from 2016 were produced by linearly interpolating the level of intervention coverage from baseline (which is country specific in countries with a linked dataset or the sample median in countries without a linked dataset) to reach the national level of current utilization as the ideal in 2020.

### Analysis plan

**Fig 2:**
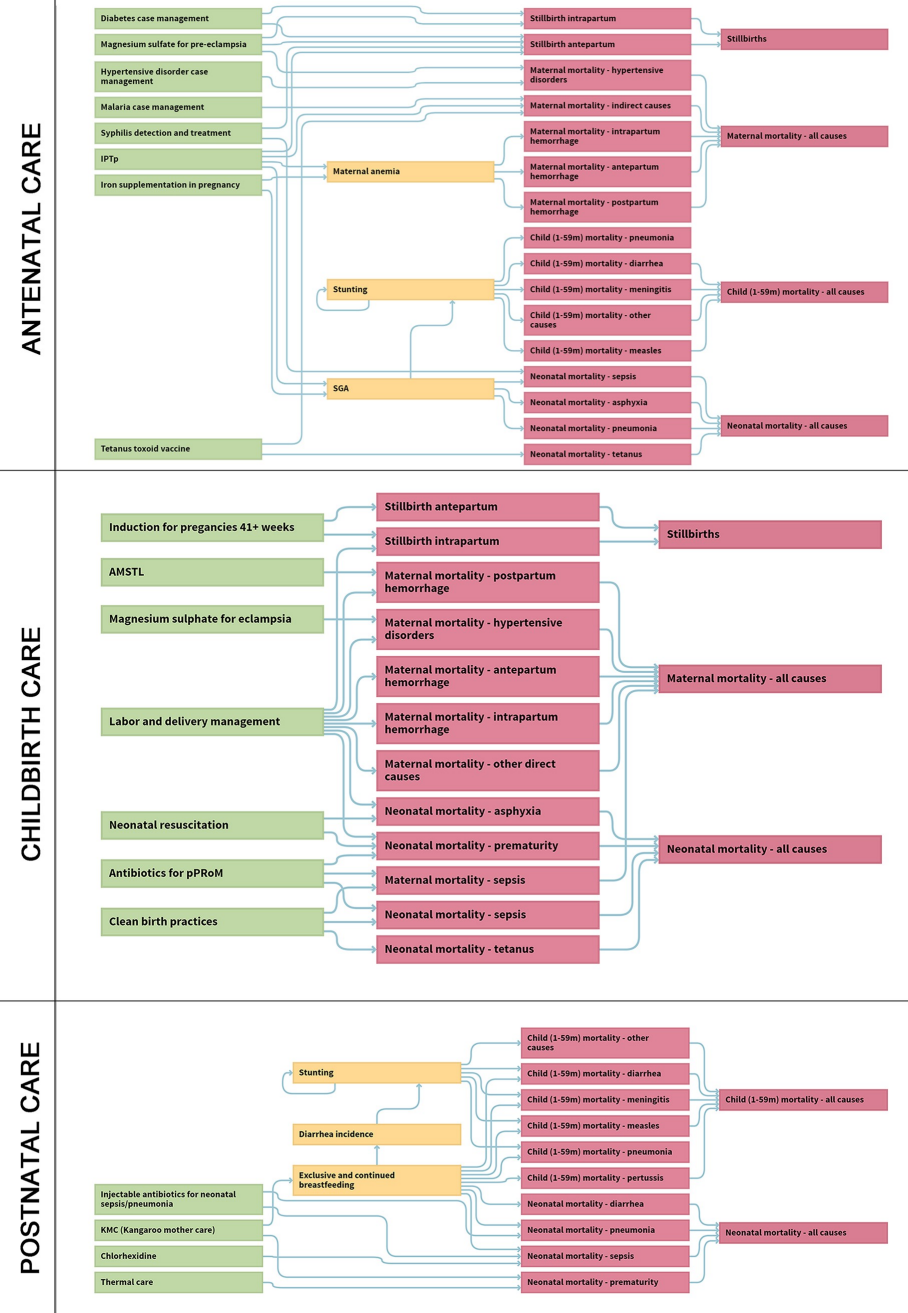
Impact pathways for the LiST (Version 5.67). Interventions provided during antenatal, childbirth, and postnatal care are shown with associated risk factors and mortality outcomes. AMTSL, active management of the third stage of labor; IPTp, Intermittent preventive treatment in pregnancy; LiST, Lives Saved Tool; pPRoM, preterm premature rupture of the membranes; SGA, small for gestational age.

For all other interventions included in the LiST model that were not relevant for this “quality of care improvement” scenario, coverage was held constant and did not increase, so these interventions did not contribute to reductions in mortality or morbidity. Examples include childhood immunizations, oral rehydration solution (ORS), and ownership of insecticide-treated bednets. Fig 2 presents the impact pathways for each set of grouped interventions, and the key outcomes of interest were changes in estimated rates for maternal and neonatal mortality and stillbirths, as well as the specific contribution of individual interventions to the total number of lives saved. Health impact was quantified as the number of “lives saved” in each country-specific model and all results were ultimately presented in aggregate as a global total representing the study period to 2020.

## Results

### Impact on mortality

**Fig 3:**
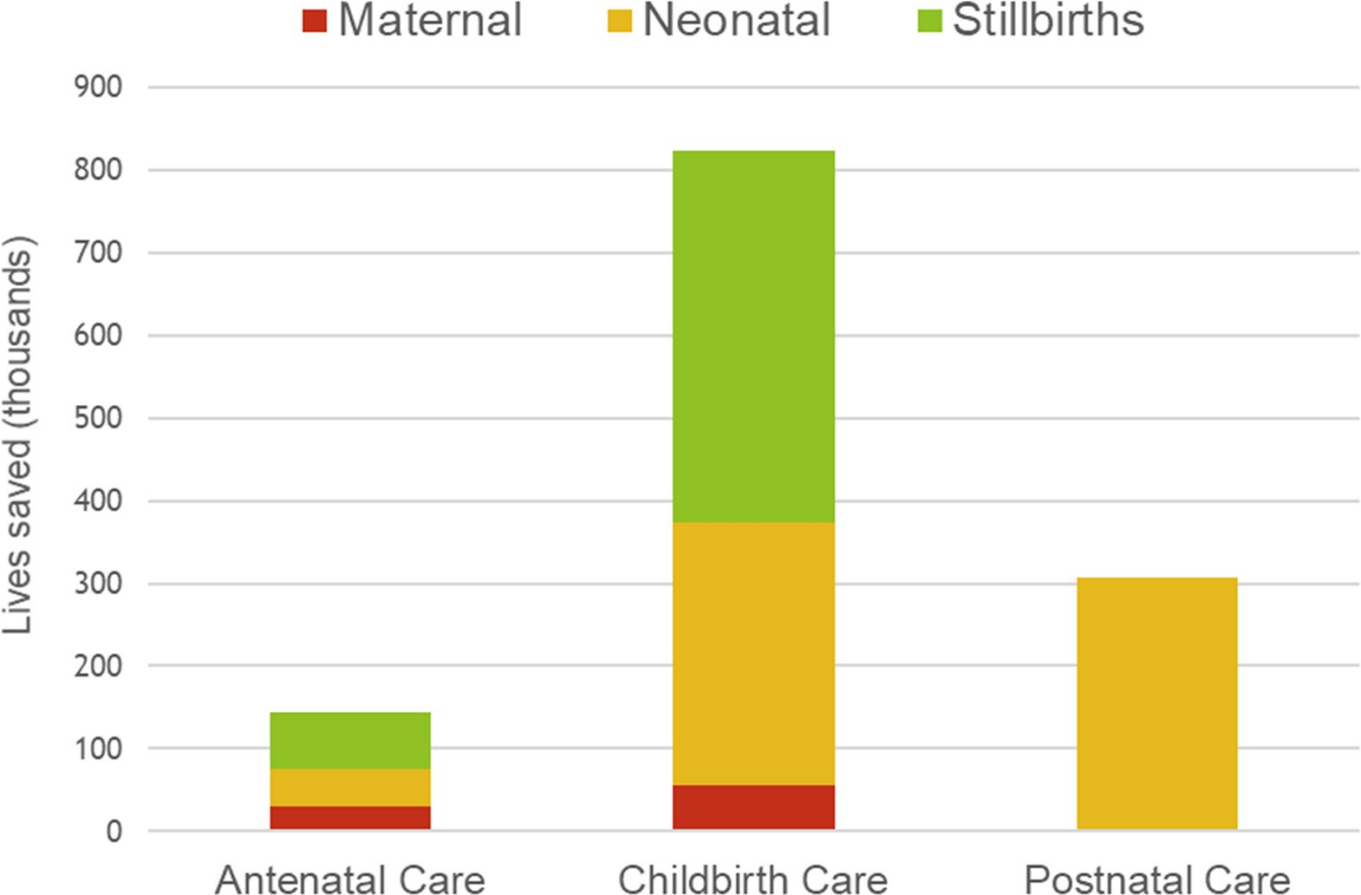
Bar graph of estimated impact in 81 LMICs, with 2020 as the target year. The total number of maternal (tan) and neonatal (orange) lives saved and stillbirths prevented (green) organized by the timing of delivery for the evidence-based intervention. LMIC, low- and middle-income country.

Our analysis focused on modeling “what if” well-functioning health systems could ensure high-quality care to merely provide a subset of 19 evidence-based maternal and neonatal interventions to those who are currently accessing or utilizing the existing service platforms. Present levels of utilization were found to be low overall, with an average of 60% (6%–96%, minimum–maximum) of women reportedly attending four or more antenatal visits and 66% (9%–99%, minimum–maximum) of deliveries occurring in a health facility, based upon national reports. Despite incorporating these low levels of utilization as the modest targets for 2020, we estimated a substantial reduction in the aggregate global sum of maternal deaths (28% decrease), neonatal deaths (28% decrease), and stillbirths (22% decrease) in the final year compared to a scenario without any change or improvement in the quality of care. In our representative sample of 81 high-burden countries, a total of 86,000 (range, 77,800–92,400) maternal, 0.67 million (range, 0.59 million–0.75 million) neonatal lives could be saved and 0.52 million (range, 0.48 million–0.55 million) stillbirths could be prevented in 2020 if populations in LMIC settings maintained the current levels of health system use but the standard for quality of clinical care was raised (Fig 3).

**Fig 4:**
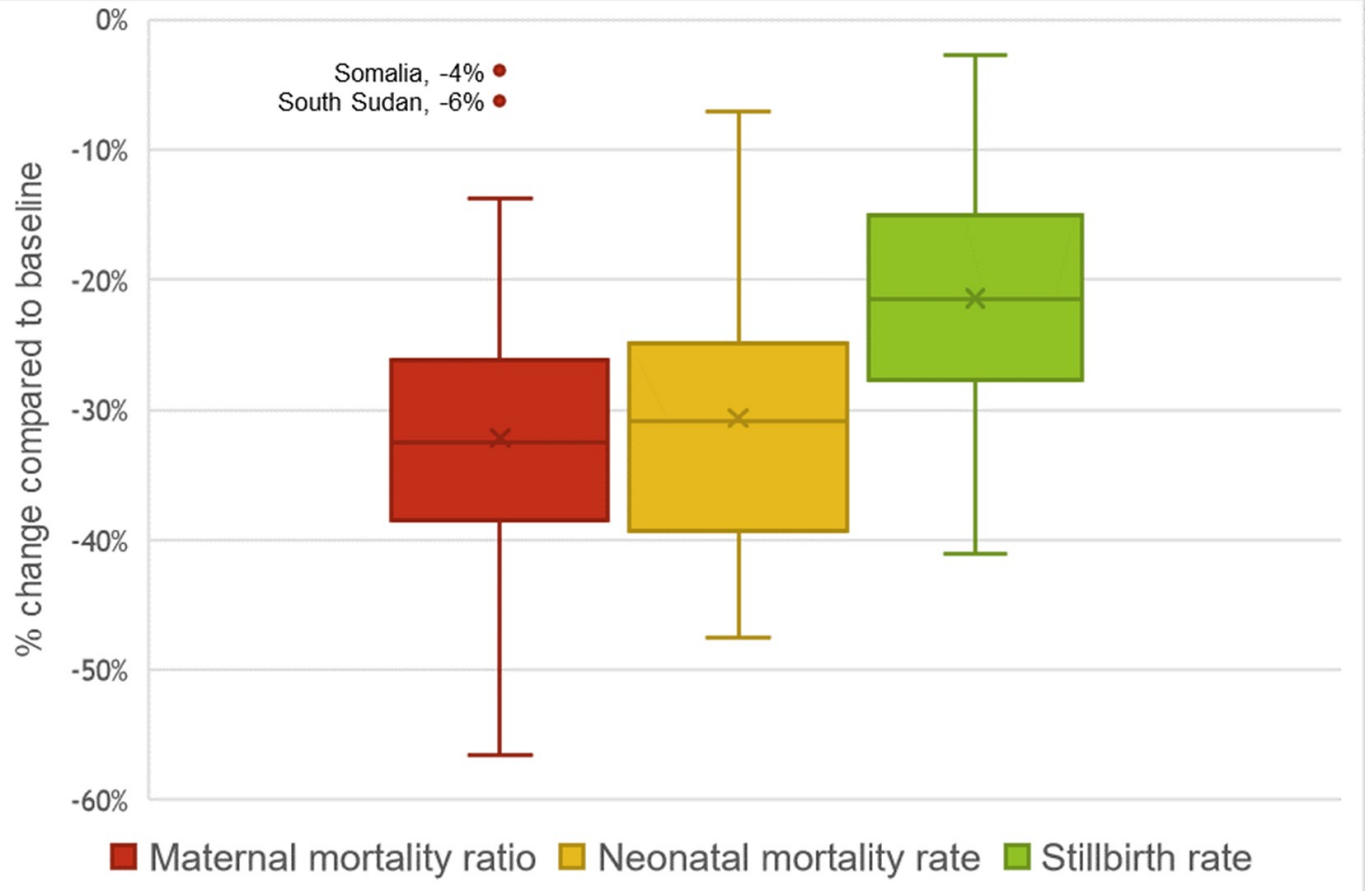
Boxplot of estimated mortality change quantified in 81 LMICs comparing 2020 (endline) to 2016 (baseline). Bars represent the 25th, 50th (middle), and 75th percentiles for the percent change in MMR, neonatal mortality rate, and stillbirth rate. The outliers include Somalia and South Sudan, which were estimated to have very modest reductions in MMR. LMIC, low- and middle-income country; MMR, maternal mortality ratio.

Ensuring a minimum threshold of quality for this set of MNCH interventions would also produce declines in estimated mortality rates. On average, maternal mortality ratio (MMR) would drop 32%, neonatal mortality rate would decrease 31%, and the stillbirth rate would drop 21% according to the modeled projections for these 81 countries (Fig 4). For maternal mortality, the change in MMR was minimal for the two countries of Somalia and South Sudan, where the estimated reductions were −4% and −6%, respectively. Accordingly, the country-specific targets set for 2020 based upon current utilization were also the lowest for these two countries (Somalia: 6% ANC4+, 9% HFD; South Sudan: 17% ANC4+, 12% HFD).

### Contribution by intervention

Of the life-saving interventions that were examined, improvements to close the quality gap for key interventions provided at or around the critical time of childbirth would produce the greatest benefit. Interventions during this period accounted for 64% of the impact overall (87% of prevented stillbirths, 47% of neonatal deaths, and 67% of maternal deaths), which exceeded benefits quantified for the antenatal or postnatal periods.

## Discussion

### Main findings

In this study, we created linear and deterministic models to examine the global impact of low-quality or inadequate care in a representative sample of 81 LMICs with recent national-level data. We quantified potential declines in mortality and found that gains would be sizeable if baseline levels of a focused package of life-saving interventions were set to reach higher levels, with effective coverage equivalent to currently reported utilization. Our analysis used a novel linking approach to combine health facility datasets, which provide an assessment of readiness, with household surveys, which report on patterns of utilization around the same time period. This ecological approach is advantageous to estimate baseline coverage for important interventions that cannot be tracked through the standard approach, which relies on participant self-reports in household surveys. This set of country-specific models, which represents a large majority of the global burden, found that scale-up of high-quality care for selected MNCH interventions would be invaluable.

### Strengths and limitations

The strengths of our study are based upon the analysis of a large, population-based, representative sample of countries (*n* = 81) where most of the maternal and neonatal deaths and stillbirths are occurring. Using a mathematical model, our quantification of the benefits of eliminating poor-quality care is based upon recent country-level data collected with health facility and household surveys. The estimates of health impact presented here focused exclusively on mothers, newborns, and stillbirths as the direct beneficiaries of the life-saving interventions we modeled, and we did not account for any of the broader impact if adverse consequences known to be associated with these fatalities [24–26] are averted. Although processes of care were not explicitly examined in this work, coverage of practices such as early initiation or exclusive breastfeeding, for example, may increase if better trained medical staff or enhanced supervision is available, as the health workforce is acknowledged to be a critical pillar of quality improvement [27]. Lastly, our scope did not include all MNCH interventions but was selectively narrow to only certain antenatal, childbirth, or postnatal interventions that could be influenced by improvements in structural inputs in a health facility context. Because the types of indirect effects mentioned above may not have been fully captured, our totals are likely to be underestimates of the true global and societal rewards if well-equipped facilities with skilled health workers provided high-quality care at the right time on a population level.

Although standardized household surveys can assess coverage for a limited set of MNCH interventions [24], some bias is associated with self-report [25], and not all interventions can be tracked with this approach. Furthermore, coverage indicators for some newborn interventions presently lack consensus definitions. Standard metrics have yet to be developed and need to be validated before measurement efforts can be implemented at scale and global progress of newborn interventions can be effectively monitored [26]. Acknowledging that gaps such as these have yet to be addressed and tracking inputs for improving quality of care is complex, our approach presents an accessible snapshot of known, evidence-based interventions that span across the continuum of care. As is generally the case, this type of global analysis offers a valuable alternative because conducting an evaluation of the entire operational framework developed for the monitoring and evaluation of each country’s health system [27] is not feasible.

"Our estimates can only be as valid as the data on which they are based” [16]. The limitations of our approach center around inputs for the model, which include assumptions about the efficacy of interventions and MNCH indicators from household surveys. As a dynamic representation of current knowledge about MNCH best practices, the LiST model relies on up-to-date Cochrane reviews, meta-analyses, or Delphi estimation to reflect how effective an intervention would be to reduce cause-specific mortality. The lingering uncertainty about antenatal corticosteroid use in low-resource settings, for example [28], highlights the inherent vulnerability of using an evidence-based platform, because it must change and evolve, by definition. As new findings surface or recommendations change, assumptions need to be adjusted, and projected estimates may be subject to change as a result.

Another caveat is related to the availability of country-specific data collected through population-based surveys. Typically conducted in rounds, DHS and MICS provide national-level data every 3–5 years, with the two survey programs applying different reference periods for calculating MNCH coverage indicators [29]. Our cross-sectional approach uniformly designated 2016 as the baseline year to standardize all models, with each country-specific model incorporating indicators about health system utilization (i.e., ANC4+ and HFD) from the most recent DHS or MICS dataset available. This means that the period when fieldwork was conducted (i.e., measurement occurred) does vary across the 81 countries. To limit misinterpretation due to this information bias, estimates were presented as global totals for the representative sample in 2020.

These findings underscore the importance of addressing the considerable burden caused by extant poor- or low-quality health services that are currently available and being provided for pregnant women and newborns in LMICs. Potential reductions in maternal and neonatal mortality and stillbirths are substantial if changes can be made to ensure adequate quality for the modest subset of life-saving antenatal, intrapartum, and postnatal interventions included in this analysis. Although the “quality chasm” for maternal, newborn, and child healthcare in these low-resource settings appears daunting [30], our work highlights the first benchmark of preliminary gains that could be anticipated if the standards of quality for clinical care were established as the overarching top priority and health systems were redesigned or strengthened to deliver consistent and high-quality evidence-based care, first and foremost, to those already seeking care and readily on hand.

Our modeling presents a conservative scenario, with the level of quality being raised to only match currently reported patterns of utilization. In fact, the benefits reaped at an individual or global level could be far greater if improved quality of care influences care-seeking patterns and subsequently drives greater demand or increases utilization in the low-resource environment of LMICs. Outreach efforts, demand creation, or expansion to provide a wider range of advanced medical services are valuable initiatives that must be incorporated as stepping-stones when universal healthcare and improving health for all population subgroups are milestones on the horizon. Although a two-pronged approach dually targeting quality of care and utilization holds the most promise for driving synergistic change, this analysis shows that merely providing a core package of MNCH services during existing health system contacts would be meaningful.

An inefficient healthcare system may be criticized as being frustratingly inadequate or cumbersome, but poor-quality care can be dangerously harmful at its worst if timely and skilled medical care is not being properly provided during the appropriate window or opportunity [27,31]. Population health suffers, and indicators do not markedly improve or may even worsen among those being served despite investment in and expenditures of precious limited resources. Analyses such as the one we have presented do not offer an exhaustive roadmap to guide all future action but highlight which next steps are most urgently needed or which priorities should be prominent. As more data become widely available from health facility surveys or accessible through global monitoring efforts, contextual factors and dominant patterns may be better understood at the national or subnational levels, where significant disparities may exist for both utilization [32] and quality of healthcare components [10]. Clinical reporting and civil registration systems may be developed or bolstered to serve as consistent, complete, and coordinated data sources for frequent tracking at a lower level [33]. Building and strengthening these components of a robust health system are therefore not optional features but the required cornerstones to ensure that timely progress can be made toward the SDG ideal of “good health and well-being” universally for all the world’s people.

## Supporting information

S1 RECORD Checklist RECORD checklist. (PDF)

S1 Table LiST parameters and data sources (Version 5.67). LiST, Lives Saved Tool.
(PDF)

S2 Table Listing of countries included in the analysis. (PDF)

S1 Text HELP manual for LiST—Coverage excerpt (Version 5.67). LiST, Lives Saved Tool.
(PDF)

## Abbreviations

AMTSL: active management of the third stage of labor
ANC4+: proportion of women who attended four or more antenatal visits
DHS: Demographic and Health Surveys
HFD: percentage of births delivered in a health facility
IMPAC: Integrated Management of Pregnancy and Childbirth
IPTp: Intermittent preventive treatment in pregnancy
LiST: Lives Saved Tool
LMIC: low- and middle-income country
MICS: Multiple Indicator Cluster Surveys
MMR: maternal mortality ratio
MNCH: maternal, neonatal, and child health
ORS: oral rehydration solution
pPRoM: preterm premature rupture of the membranes
RDT: rapid diagnostic test
RPR: rapid plasma reagin
SARA: Service Availability and Readiness Assessment
SDG: Sustainable Development Goal
SGA: small for gestational age
SPA: Service Provision Assessment
VDRL: venereal disease research laboratory
WHO: World Health Organization

## References

[1] World Health Organization. Everybody business: strengthening health systems to improve health outcomes: WHO’s framework for action. Geneva; 2007.

[2] Victora CG, Requejo JH, Barros AJ, Berman P, Bhutta Z, Boerma T, et al Countdown to 2015: a decade of tracking progress for maternal, newborn, and child survival. Lancet. 2016;387(10032):2049–59. 10.1016/S0140-6736(15)00519-X 26477328 PMC7613171

[3] Countdown to 2030 Collaboration. Countdown to 2030: tracking progress towards universal coverage for reproductive, maternal, newborn, and child health. Lancet. 2018;391(10129):1538–48. 10.1016/S0140-6736(18)30104-1 29395268

[4] Montagu D, Sudhinaraset M, Diamond-Smith N, Campbell O, Gabrysch S, Freedman L, et al Where women go to deliver: understanding the changing landscape of childbirth in Africa and Asia. Health Policy Plan. 2017;32(8):1146–52. 10.1093/heapol/czx060 28541422 PMC5886217

[5] Miller S, Abalos E, Chamillard M, Ciapponi A, Colaci D, Comande D, et al Beyond too little, too late and too much, too soon: a pathway towards evidence-based, respectful maternity care worldwide. Lancet. 2016;388(10056):2176–92. 10.1016/S0140-6736(16)31472-6 27642019

[6] Bhutta ZA, Das JK, Bahl R, Lawn JE, Salam RA, Paul VK, et al Can available interventions end preventable deaths in mothers, newborn babies, and stillbirths, and at what cost? Lancet. 2014;384(9940):347–70. 10.1016/S0140-6736(14)60792-3 24853604

[7] de Bernis L, Kinney MV, Stones W, Ten Hoope-Bender P, Vivio D, Leisher SH, et al Stillbirths: ending preventable deaths by 2030. Lancet. 2016;387(10019):703–16. 10.1016/S0140-6736(15)00954-X 26794079

[8] Leslie HH, Hirschhorn LR, Marchant T, Doubova SV, Gureje O, Kruk ME. Health systems thinking: A new generation of research to improve healthcare quality. PLoS Med. 2018;15(10):e1002682 10.1371/journal.pmed.1002682 30376581 PMC6207294

[9] Kruk ME, Pate M, Mullan Z. Introducing The Lancet Global Health Commission on High-Quality Health Systems in the SDG Era. Lancet Glob Health. 2017;5(5):e480–e1. 10.1016/S2214-109X(17)30101-8 28302563

[10] Allen E, Schellenberg J, Berhanu D, Cousens S, Marchant T. Associations between increased intervention coverage for mothers and newborns and the number and quality of contacts between families and health workers: An analysis of cluster level repeat cross sectional survey data in Ethiopia. PLoS ONE. 2018;13(8):e0199937 10.1371/journal.pone.0199937 30071026 PMC6071969

[11] Gage AD, Kruk ME, Girma T, Lemango ET. The know-do gap in sick child care in Ethiopia. PLoS ONE. 2018;13(12):e0208898 10.1371/journal.pone.0208898 30540855 PMC6291134

[12] Tuncalp O, Were WM, MacLennan C, Oladapo OT, Gulmezoglu AM, Bahl R, et al Quality of care for pregnant women and newborns-the WHO vision. BJOG. 2015;122(8):1045–9. 10.1111/1471-0528.13451 25929823 PMC5029576

[13] Donabedian A. The quality of care. How can it be assessed? JAMA. 1988;260(12):1743–8. 10.1001/jama.260.12.1743 3045356

[14] Akachi Y, Kruk ME. Quality of care: measuring a neglected driver of improved health. Bull World Health Organ. 2017;95(6):465–72. 10.2471/BLT.16.180190 28603313 PMC5463815

[15] Bryce J, Arnold F, Blanc A, Hancioglu A, Newby H, Requejo J, et al Measuring coverage in MNCH: new findings, new strategies, and recommendations for action. PLoS Med. 2013;10(5):e1001423 10.1371/journal.pmed.1001423 23667340 PMC3646206

[16] Jones G, Steketee RW, Black RE, Bhutta ZA, Morris SS, Bellagio Child Survival Study Group. How many child deaths can we prevent this year? Lancet. 2003;362(9377):65–71. 10.1016/S0140-6736(03)13811-1 12853204

[17] Walker N, Tam Y, Friberg IK. Overview of the Lives Saved Tool (LiST). BMC Public Health. 2013;13 Suppl 3:S1. 10.1186/1471-2458-13-S3-S1 PMC3847271 24564438

[18] Ng M, Fullman N, Dieleman JL, Flaxman AD, Murray CJ, Lim SS. Effective coverage: a metric for monitoring Universal Health Coverage. PLoS Med. 2014;11(9):e1001730 10.1371/journal.pmed.1001730 25243780 PMC4171091

[19] Boschi-Pinto C, Young M, Black RE. The Child Health Epidemiology Reference Group reviews of the effectiveness of interventions to reduce maternal, neonatal and child mortality. Int J Epidemiol. 2010;39 Suppl 1:i3–6. 20348123 10.1093/ije/dyq018 PMC2845857

[20] You D, Hug L, Ejdemyr S, Idele P, Hogan D, Mathers C, et al Global, regional, and national levels and trends in under-5 mortality between 1990 and 2015, with scenario-based projections to 2030: a systematic analysis by the UN Inter-agency Group for Child Mortality Estimation. Lancet. 2015;386(10010):2275–86. 10.1016/S0140-6736(15)00120-8 26361942

[21] Blencowe H, Cousens S, Jassir FB, Say L, Chou D, Mathers C, et al National, regional, and worldwide estimates of stillbirth rates in 2015, with trends from 2000: a systematic analysis. Lancet Glob Health. 2016;4(2):e98–e108. 10.1016/S2214-109X(15)00275-2 26795602

[22] Kanyangarara M, Chou VB. Linking household surveys and health facility assessments to estimate intervention coverage for the Lives Saved Tool (LiST). BMC Public Health. 2017;17(Suppl 4):780 10.1186/s12889-017-4743-4 29143639 PMC5688485

[23] Kanyangarara M, Chou VB, Creanga AA, Walker N. Linking household and health facility surveys to assess obstetric service availability, readiness and coverage: evidence from 17 low- and middle-income countries. J Glob Health. 2018;8(1):010603 10.7189/jogh.08.010603 29862026 PMC5963736

[24] Burden C, Bradley S, Storey C, Ellis A, Heazell AE, Downe S, et al From grief, guilt pain and stigma to hope and pride—a systematic review and meta-analysis of mixed-method research of the psychosocial impact of stillbirth. BMC Pregnancy Childbirth. 2016;16:9 10.1186/s12884-016-0800-8 26785915 PMC4719709

[25] Moucheraud C, Worku A, Molla M, Finlay JE, Leaning J, Yamin A. Consequences of maternal mortality on infant and child survival: a 25-year longitudinal analysis in Butajira Ethiopia (1987–2011). Reprod Health. 2015;12 Suppl 1:S4. 10.1186/1742-4755-12-S1-S4 PMC4423767 26001059

[26] Molla M, Mitiku I, Worku A, Yamin A. Impacts of maternal mortality on living children and families: A qualitative study from Butajira, Ethiopia. Reprod Health. 2015;12 Suppl 1:S6. 10.1186/1742-4755-12-S1-S6 PMC4423766 26001276

[27] Kruk ME, Gage AD, Arsenault C, Jordan K, Leslie HH, Roder-DeWan S, et al High-quality health systems in the Sustainable Development Goals era: time for a revolution. Lancet Glob Health. 2018;6(11):e1196–e252. 10.1016/S2214-109X(18)30386-3 30196093 PMC7734391

[28] Vogel JP, Oladapo OT, Pileggi-Castro C, Adejuyigbe EA, Althabe F, Ariff S, et al Antenatal corticosteroids for women at risk of imminent preterm birth in low-resource countries: the case for equipoise and the need for efficacy trials. BMJ Glob Health. 2017;2(3):e000398 10.1136/bmjgh-2017-000398 29082019 PMC5656119

[29] Hancioglu A, Arnold F. Measuring coverage in MNCH: tracking progress in health for women and children using DHS and MICS household surveys. PLoS Med. 2013;10(5):e1001391 10.1371/journal.pmed.1001391 23667333 PMC3646216

[30] Persson LA. Bridging the quality chasm in maternal, newborn, and child healthcare in low- and middle-income countries. PLoS Med. 2017;14(12):e1002465 10.1371/journal.pmed.1002465 29232389 PMC5726613

[31] Kruk ME, Gage AD, Joseph NT, Danaei G, Garcia-Saiso S, Salomon JA. Mortality due to low-quality health systems in the universal health coverage era: a systematic analysis of amenable deaths in 137 countries. Lancet. 2018;392(10160):2203–12. 10.1016/S0140-6736(18)31668-4 30195398 PMC6238021

[32] Saad-Haddad G, DeJong J, Terreri N, Restrepo-Mendez MC, Perin J, Vaz L, et al Patterns and determinants of antenatal care utilization: analysis of national survey data in seven countdown countries. J Glob Health. 2016;6(1):010404 10.7189/jogh.06.010404 27231540 PMC4871063

[33] Bhattacharya AA, Umar N, Audu A, Felix H, Allen E, Schellenberg JRM, et al Quality of routine facility data for monitoring priority maternal and newborn indicators in DHIS2: A case study from Gombe State, Nigeria. PLoS ONE. 2019;14(1):e0211265 10.1371/journal.pone.0211265 30682130 PMC6347394

