# Supplementary material for: Estimating the global impact of poor quality of care on maternal and neonatal outcomes in 81 low- and middle-income countries: A modeling study
Source: PLoS Med. 2019 Dec 18;16(12):e1002990. doi: 10.1371/journal.pmed.1002990 (PMC6919595; doi:10.1371/journal.pmed.1002990)
Supplement: S1 Table — LiST, Lives Saved Tool. (PDF) [file pmed.1002990.s002.pdf]

S1 TABLE: Lives Saved Tool parameters and data sources (Version 5.67)

Modeling Parameter	Data Source (default)
**MORTALITY**	
**Child/neonatal**	
Child and neonatal mortality	United Nations Inter-agency Group for Child Mortality Estimation (UN IGME) [1]
Cause of death distribution	Liu et al. (2016) [2]
**Maternal**	
Maternal mortality	WHO, UNICEF, UNFPA, World Bank Group and the United Nations Population Division. Trends in maternal mortality: 1990 to 2015. [3] http://www.who.int/reproductivehealth/publications/monitoring/maternal-mortality-2015/en/
Cause of death distribution	Say et al. (2014) [4]
**Stillbirths**	
Stillbirth rates	WHO estimates for years 2000-2015 http://datacompass.lshtm.ac.uk/115/
Percent by proximate causes *(i.e. antepartum, intrapartum)*	Lawn et al. (2016) [5]
**HEALTH STATUS AND COVERAGE**	
Nutritional deficiencies	Demographic and Health Surveys (DHS) and Multiple Indicator Cluster Surveys (MICS)
Anthropometry indicators	Demographic and Health Surveys (DHS) and Multiple Indicator Cluster Surveys (MICS)
Intervention coverage	Demographic and Health Surveys (DHS) and Multiple Indicator Cluster Surveys (MICS)

[1] UNICEF W, World Bank Group and United Nations, Levels & Trends in Child Mortality: Report 2017, Estimates developed by the United Nations Inter-agency Group for Child Mortality Estimation. New York, 2017.

[2] Liu L, Oza S, Hogan D, et al. Global, regional, and national causes of under-5 mortality in 2000–15: an updated systematic analysis with implications for the Sustainable Development Goals. Lancet (London, England) 2016;388(10063):3027-35. doi: 10.1016/S0140-6736(16)31593-8.

[3] WHO, UNICEF, UNFPA, World Bank Group and the United Nations Population Division. Trends in maternal mortality: 1990 to 2015. http://www.who.int/reproductivehealth/publications/monitoring/maternal-mortality-2015/en/

[4] Say L, Chou D, Gemmill A, et al. Global causes of maternal death: A WHO systematic analysis. Lancet Global Health 2014; 2(6): e323-33. http://www.ncbi.nlm.nih.gov/pubmed/25103301.

[5] Lawn JE, Blencowe H, Waiswa P, et al. Stillbirths: rates, risk factors, and acceleration towards 2030. Lancet 2016; 387: 587-603. http://www.ncbi.nlm.nih.gov/pubmed/26794078. (Supplementary appendix.)
