# Supplementary material for: Estimating the global impact of poor quality of care on maternal and neonatal outcomes in 81 low- and middle-income countries: A modeling study
Source: PLoS Med. 2019 Dec 18;16(12):e1002990. doi: 10.1371/journal.pmed.1002990 (PMC6919595; doi:10.1371/journal.pmed.1002990)
Supplement: S2 Table — (PDF) [file pmed.1002990.s003.pdf]

S2 TABLE: Listing of countries included in the analysis

Countdown to 2030 Country	iso3	Group 1	Group 2	Most recent household survey	Linked Dataset
Afghanistan	AFG	YES		DHS 2015	
Algeria	DZA	YES		MICS 2012	
Angola	AGO	YES		DHS 2015	
Azerbaijan	AZE		YES	DHS 2006	
Bangladesh	BGD	YES		DHS 2014	X
Benin	BEN	YES		MICS 2014	X
Bhutan	BTN		YES	MICS 2010	
Bolivia	BOL	YES		DHS 2008	
Botswana	BWA		YES	Family Health Survey 2007	
Burkina Faso	BFA	YES		DHS 2010	X
Burundi	BDI	YES		DHS 2016	
Cambodia	KHM	YES		DHS 2014	
Cameroon	CMR	YES		MICS 2014	
Central African Republic	CAF	YES		MICS 2010	
Chad	TCD	YES		DHS 2014	
Comoros	COM		YES	DHS 2012	
Congo, Rep.	COG	YES		MICS 2014	
Congo, Dem. Rep.	COD	YES		DHS 2013	X
Côte d'Ivoire	CIV	YES		DHS 2011	
Djibouti	DJI		YES	MICS 2006	
Dominican Republic	DOM		YES	MICS 2014	
Equatorial Guinea	GNQ		YES	DHS 2011	
Eritrea	ERI	YES		DHS 2002	
Ethiopia	ETH	YES		DHS 2016	
Gabon	GAB		YES	DHS 2012	
Gambia, The	GMB	YES		DHS 2013	
Ghana	GHA	YES		DHS 2014	
Guatemala	GTM		YES	DHS 2014	
Guinea	GIN	YES		DHS 2012	
Guinea-Bissau	GNB		YES	MICS 2014	
Guyana	GUY		YES	MICS 2014	
Haiti	HTI	YES		DHS 2012	X
Honduras	HND		YES	DHS 2011	
India	IND	YES		NFHS 2015	
Indonesia	IDN	YES		DHS 2012	
Iraq	IRQ	YES		MICS 2011	
Jamaica	JAM		YES	MICS 2011	

Kenya	KEN	YES		DHS 2014	X
Korea, Dem. Rep.	PRK		YES	MICS 2009	
Kyrgyz Republic	KGZ		YES	MICS 2014	
Lao PDR	LAO		YES	MICS 2011	
Lesotho	LSO		YES	DHS 2014	
Liberia	LBR	YES		DHS 2013	
Madagascar	MDG	YES		DHS 2008	
Malawi	MWI	YES		DHS 2015	X
Mali	MLI	YES		MICS 2015	
Mauritania	MRT	YES		MICS 2015	X
Morocco	MAR	YES		DHS 2003	
Mozambique	MOZ	YES		DHS 2011	
Myanmar	MMR	YES		DHS 2015	
Namibia	NAM		YES	DHS 2013	X
Nepal	NPL	YES		DHS 2016	X
Nicaragua	NIC		YES	RHS 2006	
Niger	NER	YES		DHS 2012	
Nigeria	NGA	YES		MICS 2016	
Pakistan	PAK	YES		DHS 2012	
Panama	PAN		YES	MICS 2013	
Papua New Guinea	PNG	YES		DHS 2006	
Paraguay	PRY		YES	RHS 2008	
Philippines	PHL	YES		DHS 2013	
Rwanda	RWA	YES		DHS 2014	X
Senegal	SEN	YES		DHS 2016	X
Sierra Leone	SLE	YES		DHS 2013	X
Solomon Islands	SLB		YES	DHS 2006	
Somalia	SOM	YES		MICS 2006	
South Africa	ZAF	YES		DHS 2003	
South Sudan	SSD	YES		MICS 2010	
Sudan	SDN	YES		MICS 2014	
Suriname	SUR		YES	MICS 2010	
Swaziland	SWZ		YES	MICS 2014	
Tajikistan	TJK		YES	DHS 2012	
Tanzania	TZA	YES		DHS 2015	X
Timor-Leste	TLS		YES	DHS 2016	
Togo	TGO	YES		DHS 2013	X
Turkmenistan	TKM		YES	MICS 2015	
Uganda	UGA	YES		DHS 2016	X
Uzbekistan	UZB		YES	MICS 2006	

Venezuela, RB	VEN	YES		UNICEF SOWC 2009-2013 estimate	
Yemen, Rep.	YEM	YES		DHS 2013	
Zambia	ZMB	YES		DHS 2013	
Zimbabwe	ZWE	YES		DHS 2015	X
**TOTAL**		**53**	**28**		**17**

Group 1: U5MR and/or MMR above target and included in countries accounting for 95% of all U5 or maternal deaths

Group 2: U5MR and/or MMR above target but **not** included in countries accounting for 95% of all U5 or maternal deaths

(In-depth country profiles and reports are available from ***Countdown to 2030***)
