# Supplementary material for: Estimating the global impact of poor quality of care on maternal and neonatal outcomes in 81 low- and middle-income countries: A modeling study
Source: PLoS Med. 2019 Dec 18;16(12):e1002990. doi: 10.1371/journal.pmed.1002990 (PMC6919595; doi:10.1371/journal.pmed.1002990)
Supplement: S1 Text — LiST, Lives Saved Tool. (PDF) [file pmed.1002990.s004.pdf]

Lives Saved Tool (LiST)
HELP Manual

Coverage excerpt
Version 5.67
Released 10 May 2018

Coverage

This section describes the types of interventions which are available within LiST for modification. Each type is characterized by either the time in which the intervention is delivered (i.e., during pregnancy) or by the mode of activity (i.e., vaccines or curative). All items which fall under the categories of breastfeeding, preventive, vaccines, and curative occur after birth. Select any one of the types/time periods for more information on all interventions which are included.

To enter coverage data:

1. Click on the tab for the editor screen that you wish to work in, to bring that screen to the forefront.

2. Review the default values listed for each child health intervention for all years displayed.

3. Edit the coverage targets for future years past the first year of intervention manually if you have datayou feel is more accurate than what is listed. If you would like to make this first projection a "do nothing" scenario (or a theoretical counterfactual for your intended coverage changes), where the intervention coverage is by default held constant from the first year of intervention, do not change the default values after the first year of intervention. Click "Ok" and save the projection through the "Home" tab of the Spectrum menu. Then, proceed to reviewing effectiveness values.

4. Edit the coverage values from the first year of intervention to the target year. **By changing the target, you will be able to project the impact upon child survival in your country.** Most often, users set a target for the final year of the projection (2015 in the case of the MDGs) and interpolate between the base year and the target year.

5. An easy way to change this coverage is to set the coverage in the target year and highlight from the first year of intervention to the target year. Then right click and scroll to "interpolate". There are four different interpolate options. Choose the one that best suits your data. Use the duplicate function if you would like several adjoining data boxes in a row or column to have the same value.

Periconceptual

Contraceptive use Folic acid supplementation/fortification Blanket iron supplementation/fortification Safe abortion services Post-abortion case management Ectopic pregnancy case management

Contraceptive use

 Definition: Coverage and effectiveness of Family Planning interventions are specified in the FamPlan module.

Folic acid supplementation/fortification

- **Definition:** Percent of women 15-49 that are taking folic acid supplements (5.0 mg folic acid per day) or have appropriate food fortification around the time of pregnancy.
- **Default data source:** Coverage data for this indicator are not typically available. Currently set at 0 for baseline; user should enter local data if possible and available.
- **Notes:** This is not the same indicator as iron/folate supplementation during pregnancy.

  The effect size used by default in LiST is for fortification of staple foods with folic acid. If the user wishes to model periconceptual folic acid supplementation instead, the effect size should be modified to 0.62. Both values are from the same source paper, cited directly below.

- **Effect size reference:** Blencowe H, Cousens S, Modell B, et al. Folic acid to reduce neonatal mortality from neural tube disorders. International Journal of Epidemiology 2010; 39(Suppl 1): i110-i121. http://www.ncbi.nlm.nih.gov/pmc/articles/PMC2845867/.

  Imdad A, Yakoob MY, Bhutta ZA. The effect of folic acid, protein energy and multiple micronutrient supplements in pregnancy on stillbirths. BMC Public Health 2011; 11(Suppl 3): S4. http://www.ncbi.nlm.nih.gov/pmc/articles/PMC3231910/.

  De-Regil LM, Pena-Rosas JP, Fernandez-Gaxiola AC, et al. Effects and safety of periconceptional oral folate supplementation for preventing birth defects. Cochrane Database Syst Rev 2015. http://www.ncbi.nlm.nih.gov/pubmed/26662928.

Blanket iron supplementation/fortification

- **Definition:** Percent of women 15-49 that are taking daily iron supplements or have appropriate food fortification around the time of pregnancy.
- **Default data source:** Coverage data for this indicator are not typically available. Currently set at 0 for baseline; user should enter local data if possible and available.
- **Notes:** This is not the same indicator as iron supplementation during pregnancy.

  The effect size used by default in LiST is for daily iron supplementation among women of reproductive age. If the user wishes to model fortification of staple foods with iron instead, the recommended effect size is 0.36 (effect of iron fortification on anemia, studies from low- and middle-income countries only, from Das et al. 2013).

- **Effect size reference:** Low MS, Speedy J, Styles CE, et al. Daily iron supplementation for improving anaemia, iron status and health in menstruating women. Cochrane Database Syst Rev 2016. http://www.ncbi.nlm.nih.gov/pubmed/27087396.

Safe abortion services

- **Definition:** Among women who get an abortion, the percent who get a safe abortion (defined as via D&C, vacuum aspiration, or medical abortion).
- **Default data source:** Sedgh G, Singh S, Shah IH, et al. Induced abortion: Incidence and trends worldwide from 1995 to 2008. Lancet 2012; 379(9816): 625-32. http://www.ncbi.nlm.nih.gov/pubmed/22264435.
- **Notes:** Country-specific estimates are not available. Regional data are being used.
- **Effect size reference:** Pollard SL, Mathai M, Walker N. Estimating the impact of interventions on causespecific maternal mortality: A Delphi approach. BMC Public Health 2013, 13(Suppl 3): S12. http://www.ncbi.nlm.nih.gov/pmc/articles/PMC3847442/.

Post-abortion case management

- **Definition:** Percent of women who have had an abortion who get the appropriate post-abortion case management at a Basic Emergency Obstetric Care (BEmOC) level.
- **Default data source:** Coverage data for this indicator are not typically available. Currently set at 0 for baseline; user should enter local data if possible and available.
- **Notes:**
- **Effect size reference:** Pollard SL, Mathai M, Walker N. Estimating the impact of interventions on causespecific maternal mortality: A Delphi approach. BMC Public Health 2013, 13(Suppl 3): S12. http://www.ncbi.nlm.nih.gov/pmc/articles/PMC3847442/.

Ectopic pregnancy case management

- **Definition:** Percent of women with an ectopic pregnancy who receive case management at a Basic Emergency Obstetric Care (BEmOC) level.
- **Default data source:** Coverage data for this indicator are not typically available. Currently set at 0 for baseline; user should enter local data if possible and available.
- **Notes:**
- **Effect size reference:** Pollard SL, Mathai M, Walker N. Estimating the impact of interventions on causespecific maternal mortality: A Delphi approach. BMC Public Health 2013, 13(Suppl 3): S12. http://www.ncbi.nlm.nih.gov/pmc/articles/PMC3847442/.

Pregnancy

Antenatal care TT - tetanus toxoid vaccination IPTp - pregnant women protected via intermittent preventive treatment of malaria Syphilis detection and treatment Calcium supplementation Multiple micronutrient supplementation Iron supplementation Balanced energy-protein supplementation Hypertensive disorders case management Diabetes case management Malaria case management MgSO4-management of preeclampsia FGR-fetal growth restriction detection and management PMTCT - Prevention of mother to child transmission of HIV

Antenatal care

- **Definition:** Percent of women who attend four or more antenatal care visits during their pregnancy (ANC 4+).

- **Default data source:** Coverage data for this indicator are drawn from DHS, MICS, and other nationally representative household surveys.

- **Notes:** This has no direct impact and is not currently displayed in the LiST editor. It is used as a component in the regression formulas used to determine the coverage of certain interventions delivered as a part of ANC. For details on the calculation of coverage of antenatal care interventions in LiST, please see this LiST Technical Note and publication by Kanyangarara and Chou, 2017.

  Kanyangara M and Chou V. Linking household surveys and health facility assessments to estimate intervention coverage for the Lives Saved Tool (LiST). BMC Public Health 2017; 17(Suppl 4):780. https://www.ncbi.nlm.nih.gov/pubmed/29143639.

TT - tetanus toxoid vaccination

- **Definition:** Percent of neonates who are protected at birth (PAB) from tetanus infection. PAB is defined as the percent of women who received two doses of tetanus toxoid during this pregnancy or ever: Received at least 2 doses, the last within 3 years; received at least three doses, the last within 5 years; received at least 4 doses, the last within 10 years; or received at least five doses during lifetime. Also known as TT2+.

- **Default data source:** WHO/UNICEF. Immunization surveillance, assessment, and monitoring. "Protected at birth" indicator (http://apps.who.int/immunization_monitoring/globalsummary/timeseries/tswucoveragepab.html). The most recent available data are through 2013.

- **Notes:** Default data can be replaced with data from DHS/MICS or other sources as appropriate.

- **Effect size reference:** Blencowe H, Lawn J, Vandelaer J, et al. Tetanus toxoid immunization to reduce mortality from neonatal tetanus. International Journal of Epidemiology 2010; 39(Suppl 1): i102-i109. http://www.ncbi.nlm.nih.gov/pmc/articles/PMC2845866/.

IPTp - pregnant women protected via intermittent preventive treatment of malaria

- **Definition:** Percent of pregnant women receiving 2+ doses of Sp/Fansidar during pregnancy.
- **Default data source:** Coverage data for this indicator are drawn from DHS, MICS, and other nationally representative household surveys. This also includes Malaria Indicator Surveys.
- **Notes:** By default, this indicator can only be modified if the national government recommends IPTp. This can be changed on the "Baseline child health status" tab of the "Health status, mortality, and economic status" menu.

  The effect is only applied to the first and second pregnancies among women who are exposed to malaria.

  If data on Sp/Fansidar is not available for a given country, data on the percentage of pregnant women sleeping under an insecticide-treated bednet (ITN) can be used instead.

- **Effect size reference:** Eisele TP, Larsen D, Steketee RW. Protective efficacy of interventions for preventing malaria mortality in children in Plasmodium falciparum endemic areas. International Journal of Epidemiology 2010; 39(Suppl 1): i88-i10. http://www.ncbi.nlm.nih.gov/pmc/articles/PMC2845865/. (The effect size for ITN/IRS is used as a proxy for IPTp.)

  Pollard SL, Mathai M, Walker N. Estimating the impact of interventions on cause-specific maternal mortality: A Delphi approach. BMC Public Health 2013; 13(Suppl 3): S12. http://www.ncbi.nlm.nih.gov/pmc/articles/PMC3847442/.

  Ishaque S, Yakoob MY, Imdad A, et al. Effectiveness of interventions to screen and manage infections during pregnancy on reducing stillbirths: A review. BMC Public Health 2011; 11(Suppl 3):
  S3. http://www.ncbi.nlm.nih.gov/pmc/articles/PMC3231903/. (The effect size for ITN usage is used as a proxy for IPTp.)

  Radeva-Petrova D, Kayentao K, Ter Kuile FO, et al. Drugs for preventing malaria in pregnant women in endemic areas: Any drug regimen versus placebo or no treatment. Cochrane Database Syst Rev 2014. http://www.ncbi.nlm.nih.gov/pmc/articles/PMC4498495/.

Syphilis detection and treatment

- **Definition:** Percent of pregnant women tested for syphilis and given treatment if needed.
- **Default data source:** Coverage data for this indicator are not typically available. As a proxy, coverage is calculated using a regression formula based on components of antenatal care available in DHS/MICS surveys (e.g., timing of first antenatal care visit, blood sample taken, urine sample taken, coverage of ANC4+). For details on this calculation, please see the LiST Technical Note and publication by Kanyangarara and Chou, 2017.

  Kanyangara M and Chou V. Linking household surveys and health facility assessments to estimate intervention coverage for the Lives Saved Tool (LiST). BMC Public Health 2017; 17(Suppl 4):780. https://www.ncbi.nlm.nih.gov/pubmed/29143639.

- **Notes:**

- **Effect size reference:** Blencowe H, Cousens S, Kamb M, et al. Lives Saved Tool supplement detection and treatment of syphilis in pregnancy to reduce syphilis related stillbirths and neonatal mortality. BMC Public Health 2011; 11(Suppl 3): S9. http://www.ncbi.nlm.nih.gov/pubmed/21501460.

Calcium supplementation

- **Definition:** Percent of pregnant women taking 1g of calcium daily.

- **Default data source:** Coverage data for this indicator are not typically available. Currently set at 0 for baseline; user should enter local data if possible and available.

- **Notes:** The effect size is applied to the percentage of the population living below $1.90 a day (a proxy for percentage of who are food insecure).This is a country-specific value found in the "Health status, mortality and economic status" tab under "Household status."

- **Effect size reference:** Jabeen M, Yakoob MY, Imdad A, et al. Impact of interventions to prevent and manage preeclampsia and eclampsia on stillbirths. BMC Public Health 2011; 11(Suppl 3): S6. http://www.ncbi.nlm.nih.gov/pmc/articles/PMC3231912/.

  Ronsmans C, Campbell O. Quantifying the fall in mortality associated with interventions related to hypertensive diseases of pregnancy. BMC Public Health 2011; 11(Suppl 3): S8. https://www.ncbi.nlm.nih.gov/pmc/articles/PMC3231914/.

  Imdad A, Jabeen A, Bhutta, ZA. Role of calcium supplementation during pregnancy in reducing risk of developing gestational hypertensive disorders: a meta-analysis of studies from developing countries. BMC Public Health 2011; 11(Suppl 3): S18. https://www.ncbi.nlm.nih.gov/pmc/articles/PMC3231891/.

Multiple micronutrient supplementation

- **Definition:** Percent of pregnant women taking a multiple micronutrient supplement daily. A multiple micronutrient supplement is defined as a supplement containing at least iron, folate, and additional vitamins/minerals.

  Default data source: Coverage data for this indicator are not typically available. Currently set at 0 for baseline; user should enter local data if possible and available.

- **Notes:** The sum of multiple micronutrient supplementation and iron supplementation cannot be greater than 100%.

- **Effect size reference:** Haider BA, Yakoob MY, Bhutta ZA. Effect of multiple micronutrient supplementation during pregnancy on maternal and birth outcomes. BMC Public Health 2011; 11(Suppl 3): S19. http://www.ncbi.nlm.nih.gov/pubmedhealth/PMH0031917/.

  Pena-Rosas JP, De-Regil LM, Garcia-Casal MN, et al. Daily oral iron supplementation during pregnancy. Cochrane Database Syst Rev 2015. http://www.ncbi.nlm.nih.gov/pubmed/26198451.

(The effect of multiple micronutrient supplementation on maternal anemia is assumed to be the same as that of iron supplementation.)

Haider BA, Bhutta ZA. Multiple-micronutrient supplementation for women during pregnancy. Cochrane Database Syst Rev 2015. http://www.ncbi.nlm.nih.gov/pubmed/26522344.

LiST Technical Note: Justification for iron and MMN supplementation effect sizes in LiST. http://livessavedtool.org/images/documents/Technical_Notes/Iron-and-MMN-effect-sizes.pdf

Iron supplementation

- **Definition:** Percent of pregnant women taking an iron supplement daily, for at least 90 days.
- **Default data source:** Coverage data for this indicator are drawn from DHS, MICS, and other nationally representative household surveys.
- **Notes:** The sum of iron supplementation and multiple micronutrient supplementation cannot be greater than 100%.
- **Effect size reference:** Yakoob MY, Bhutta ZA. Effect of routine iron supplementation with or without folic acid on anemia during pregnancy. BMC Public Health 2011; 11(Suppl 3): S21. http://www.ncbi.nlm.nih.gov/pubmedhealth/PMH0031947/.

Haider BA, Yakoob MY, Bhutta ZA. Effect of multiple micronutrient supplementation during pregnancy on maternal and birth outcomes. BMC Public Health 2011; 11(Suppl 3): S19. http://www.ncbi.nlm.nih.gov/pmc/articles/PMC3231892/.

Pena-Rosas JP, De-Regil LM, Garcia-Casal MN, et al. Daily oral iron supplementation during pregnancy. Cochrane Database Syst Rev 2015. http://www.ncbi.nlm.nih.gov/pubmed/26198451.

LiST Technical Note: Justification for iron and MMN supplementation effect sizes in LiST. http://livessavedtool.org/images/documents/Technical_Notes/Iron-and-MMN-effect-sizes.pdf

Balanced energy-protein supplementation

- **Definition:** Percent of pregnant women who are food insecure who receive balanced energy-protein (BEP) supplementation.
- **Default data source:** Coverage data for this indicator are not typically available. Currently set at 0 for baseline; user should enter local data if possible and available.

Notes: The effect size is applied to the percent of the population living on less than $1.90/day, used as a proxy for food insecurity (the "Household Status" tab of the "Health status, mortality, and economic status" menu).

- **Effect size reference:** Imdad A, Bhutta ZA. Effect of balanced protein energy supplementation during pregnancy on birth outcomes. BMC Public Health 2011; 11(Suppl 3): S17. http://www.ncbi.nlm.nih.gov/pubmedhealth/PMH0031506/.

Ota E, Hori H, Mori R, et al. Antenatal dietary education and supplementation to increase energy and protein intake. Cochrane Database Syst Rev 2015. http://www.ncbi.nlm.nih.gov/pubmed/26031211.

- **Affected fraction reference:** Jackson BD, Walker N, Heidkamp R. Metrics for Identifying Food Security
Status and the Population with Potential to Benefit from Nutrition Interventions in the Lives Saved Tool
(LiST). J Nutrition 2017, 147(11S): 2147S-2155S. https://doi.org/10.3945/jn.116.243808

Hypertensive disorders case management

- **Definition:** Percent of women receiving detection and appropriate management of moderate to severe hypertension during pregnancy.

- **Default data source:** Coverage data for this indicator are not typically available. As a proxy, coverage is calculated using a regression formula based on components of antenatal care available in DHS/MICS surveys (e.g., timing of first antenatal care visit, blood sample taken, urine sample taken, coverage of ANC4+). For details on this calculation, please see the LiST Technical Note and publication by Kanyangarara and Chou, 2017.

  Kanyangara M and Chou V. Linking household surveys and health facility assessments to estimate intervention coverage for the Lives Saved Tool (LiST). BMC Public Health 2017; 17(Suppl 4):780. https://www.ncbi.nlm.nih.gov/pubmed/29143639.

- **Notes:**

- **Effect size reference:** Pollard SL, Mathai M, Walker N. Estimating the impact of interventions on causespecific maternal mortality: A Delphi approach. BMC Public Health 2013; 13(Suppl 3): S12. http://www.ncbi.nlm.nih.gov/pmc/articles/PMC3847442/.

Diabetes case management

- **Definition:** Percent of pregnant women screened for diabetes and managed appropriately, if needed.

- **Default data source:** Coverage data for this indicator are not typically available. As a proxy, coverage is calculated using a regression formula based on components of antenatal care available in DHS/MICS surveys (e.g., timing of first antenatal care visit, blood sample taken, urine sample taken, coverage of ANC4+). For details on this calculation, please see the LiST Technical Note and publication by Kanyangarara and Chou, 2017.

  Kanyangara M and Chou V. Linking household surveys and health facility assessments to estimate intervention coverage for the Lives Saved Tool (LiST). BMC Public Health 2017; 17(Suppl 4):780. https://www.ncbi.nlm.nih.gov/pubmed/29143639.

- **Notes:** This currently only impacts stillbirths.

Effect size reference: Syed M, Javed H, Yakoob MY, et al. Effect of screening and management of diabetes during pregnancy on stillbirths. BMC Public Health 2011; 11(Suppl 3): S2. http://www.ncbi.nlm.nih.gov/pmc/articles/PMC3231893/.

Malaria case management

- **Definition:** Percent of pregnant women experiencing malaria that are appropriately managed.
- **Default data source:** Coverage data for this indicator are not typically available. As a proxy, coverage is calculated using a regression formula based on components of antenatal care available in DHS/MICS surveys (e.g., timing of first antenatal care visit, blood sample taken, urine sample taken, coverage of ANC4+). For details on this calculation, please see the LiST Technical Note and publication by Kanyangarara and Chou, 2017.

  Kanyangara M and Chou V. Linking household surveys and health facility assessments to estimate intervention coverage for the Lives Saved Tool (LiST). BMC Public Health 2017; 17(Suppl 4):780. https://www.ncbi.nlm.nih.gov/pubmed/29143639.
- **Notes:** The intervention covers the entire period between conception and six weeks after delivery.
- **Effect size reference:** Pollard SL, Mathai M, Walker N. Estimating the impact of interventions on causespecific maternal mortality: A Delphi approach. BMC Public Health 2013; 13(Suppl 3): S12. http://www.ncbi.nlm.nih.gov/pmc/articles/PMC3847442/.

$MgSO_4$- management of pre-eclampsia

- **Definition:** Percent of pregnant women with pre-eclampsia who are treated with intravenous magnesium sulfate (4-6g).
- **Default data source:** Coverage data for this indicator are not typically available. As a proxy, coverage is calculated using a regression formula based on components of antenatal care available in DHS/MICS surveys (e.g., timing of first antenatal care visit, blood sample taken, urine sample taken, coverage of ANC4+). For details on this calculation, please see the LiST Technical Note and publication by Kanyangarara and Chou, 2017.

  Kanyangara M and Chou V. Linking household surveys and health facility assessments to estimate intervention coverage for the Lives Saved Tool (LiST). BMC Public Health 2017; 17(Suppl 4):780. https://www.ncbi.nlm.nih.gov/pubmed/29143639.
- **Notes:**
- **Effect size reference:** Ronsmans C, Campbell O. Quantifying the fall in mortality associated with interventions related to hypertensive diseases of pregnancy. BMC Public Health 2011; 11(Suppl 3): S8. http://www.ncbi.nlm.nih.gov/pubmed/21501459.

  Jabeen M, Yakoob MY, Imdad A, et al. Impact of interventions to prevent and manage preeclampsia and eclampsia on stillbirths. BMC Public Health 2011; 11(Suppl 3): S6. http://www.ncbi.nlm.nih.gov/pmc/articles/PMC3231912/.

FGR - fetal growth restriction detection and management

Definition: Percent of pregnancies screened for fetal growth restriction (including BMI, fundal height, ultrasound, and/or Doppler) and managed with appropriate obstetric intervention, including early delivery, if needed.

- **Default data source:** Coverage data for this indicator are not typically available. Currently set at 0 for baseline; user should enter local data if possible and available.

- **Notes:** Based on a review by Imdad and colleagues, the impact of detection and management of fetal growth restriction was added to LiST. (Imdad A, Yakoob MY, Siddiqui S, et al. Screening and triage of intrauterine growth restriction (IUGR) in general population and high risk pregnancies: a systematic review with a focus on reduction of IUGR related stillbirths. BMC Public Health 2011, 11(Suppl 3): S1. http://www.ncbi.nlm.nih.gov/pmc/articles/PMC3231882/.) The review found that proper detection and management of IUGR (fetal growth restriction) could reduce stillbirths by 20%. However, this analysis depends on proper management, often including induced labor and Cesarean sections, that may not be available in low-resource settings. In addition, by producing earlier births, management does reduce the stillbirths, but the child of the resulting birth may be premature and appropriate management of premature babies, especially those very premature, may not be available. The users who choose to scale up FGR should be aware of these difficulties and will need to specify the effectiveness of the intervention.

- **Effect size reference:** The effect size is currently set to 0 (see above); use the "Effectiveness" menu to modify if necessary.

PMTCT - Prevention of mother to child transmission of HIV

- **Definition:** Coverage and effectiveness of PMTCT interventions are specified in the AIM module.
- **Default data source:**

Childbirth

Skilled birth attendance (SBA) Health facility delivery Unassisted delivery Assisted delivery at home Essential care BEmOC CEmOC Clean birth practices Immediate assessment and stimulation Labor and delivery management Neonatal resuscitation Antenatal corticosteroids for preterm labor Antibiotics for PPRoM $MgSO_4$-management of eclampsia AMTSL-active management of the third stage of labor Induction of labor for pregnancies lasting 41+ weeks

Within the "Childbirth" tab, LiST will automatically calculate the distributions of delivery levels and coverage levels of childbirth interventions, unless you uncheck "Allow LiST to calculate place and level of delivery" to manually change level and place of delivery or uncheck "Allow LiST to calculate intervention coverages" to manually enter coverage for childbirth interventions.

Please note that to manually edit coverage of childbirth interventions you must (after unchecking "Allow LiST to calculate intervention coverages") specify which of the childbirth interventions are available at which level of care, before proceeding to the tab for the each delivery level to enter coverage values. Coverage for each intervention can be entered as either the percentage of all deliveries, or as the percentage of that delivery level specifically. If you need to return the box where you specify the

availability of childbirth interventions, you must recheck and then uncheck the "Allow LiST to calculate intervention coverages" box. This will require you to reenter all changes made.

Skilled birth attendance (SBA)

- **Definition:** Percent of children born with a skilled attendant present, including doctors, nurses, or midwives, in a facility or at home. An SBA in the home is defined as a skilled birth attendant who delivers the infant at home without benefit of referral to a facility in case of emergency. An SBA in a facility is defined as a medically skilled attendant who has the ability and facilities needed to monitor labor progress with a partograph and detect complications. Episiotomy is available, if needed. Infection control is covered under clean birth practices.

- **Default data source:** Coverage data for this indicator are drawn from DHS, MICS, and other nationally representative household surveys.

- **Notes:** This intervention has no effect alone. It is used to calculate the coverage of home delivery interventions. See assisted deliveries at home below. The value for SBA must be greater than or equal to Facility Deliveries. (We are aware that not all deliveries at a facility have a skilled attendant. However, in the absence of additional data, we have chosen to make this assumption.)

Health facility delivery

- **Definition:** Percent of children born in a health facility.

- **Default data source:** Coverage data for this indicator are drawn from DHS, MICS, and other nationally representative household surveys.

- **Notes:** This intervention has no effect alone. It is used to calculate the estimated coverage of all childbirth care interventions in combination with skilled birth attendance.

Unassisted delivery

- **Definition:** Percent of deliveries in the home without skilled attendance.

- **Default data source:** This is calculated by subtracting the percent of births with SBA from 100% of all births.

- **Notes:** This value can only be modified by adjusting the other Facility Delivery and SBA values. The sum of the five levels of delivery must be 100%. It is assumed that there is no referral for complications and that an unassisted delivery is the highest level of care available.

Assisted delivery at home

- **Definition:** Percent of deliveries in the home with a skilled birth attendant present.

- **Default data source:** If the "Allow LiST to calculate place and level of delivery" box is checked, this is automatically calculated as SBA minus Facility Delivery.

- **Notes:** It is assumed that there is no referral to a facility for complications and that the SBA at home is the highest level of care available to the women.

Essential care

- **Definition:** Deliveries at this level of care are assumed to be in facilities that include monitoring of labor progress with a partograph, detection of complications, infection control via a clean delivery, and episiotomy if needed. For the neonate, this includes routine care practices including immediate drying, skin-to-skin contact or immediate wrapping for thermal care, and clean cord cutting.

  In LiST, the default assumption is that all Essential Care facilities provide clean birth practices, immediate assessment and stimulation of the newborn, labor and delivery management, and neonatal resuscitation. This can be modified by unchecking the box labeled "Allow LiST to calculate intervention coverages" on the "Childbirth" tab.

- **Default data source:** The percentage of deliveries at this level are calculated from Health Facility Deliveries (FacilDeliv) according to the following formula:
  - If FacilDeliv is less than 30%, then Essential care deliveries are 90% of FacilDeliv
  - If FacilDeliv is between 30% and 50%, then Essential care deliveries are 50% of FacilDeliv
  - If FacilDeliv is between 50% and 95%, then Essential care deliveries are 25% of FacilDeliv
  - If FacilDeliv are 95% or greater, then Essential care deliveries are 0% of FacilDeliv

- **Notes:** It is assumed that there is no referral to a BEmOC or CEmOC facility for complications and that Essential Care is the highest level of care available to the women.

BEmOC

- **Definition:** Deliveries at this level of care are assumed to be in facilities that meet the WHO's guidelines for Basic Emergency Obstetric and Newborn Care (BEmOC). BEmOC facilities must be able to perform seven signal functions: administer parenteral antibiotics; administer parenteral anticonvulsants; administer parenteral oxtyocics; manual removal of placenta; removal of retained products (manual vacuum aspiration); assisted vaginal delivery (with vacuum extractor or forceps); and neonatal resuscitation with bag and mask.

  In LiST, the default assumption is that all BEmOC facilities provide clean birth practices, immediate assessment and stimulation of the newborn, labor and delivery management, neonatal resuscitation, antibiotics for pPRoM, MgSO4 management of eclampsia, and active management of the third stage of labor (AMTSL). This can be modified by unchecking the box labeled "Allow LiST to calculate intervention coverages" on the "Childbirth" tab.

- **Default data source:** The percentage of deliveries at this level are calculated from Health Facility Deliveries (FacilDeliv) according to the following formula:
  - If FacilDeliv is less than 30%, then BEmOC deliveries are 0% of FacilDeliv
  - If FacilDeliv is between 30% and 50%, then BEmOC deliveries are 30% of FacilDeliv
  - If FacilDeliv is between 50% and 95%, then BEmOC deliveries are 15% of FacilDeliv
  - If FacilDeliv are 95% or greater, then BEmOC deliveries are 0% of FacilDeliv

- **Notes:** It is assumed that there is no referral to a CEmOC facility for complications and that BEmOC is the highest level of care available to the women.

CEmOC

- **Definition:** Deliveries at this level of care are assumed to be in facilities that meet the WHO's guidelines for Comprehensive Emergency Obstetric and Newborn Care (CEmOC). CEmOC facilities must be able to perform the seven signal functions of BEmOC, plus surgery (e.g. Caesarean section) and blood transfusion.

  In LiST, the default assumption is that all CEmOC facilities provide clean birth practices, immediate assessment and stimulation of the newborn, labor and delivery management, neonatal resuscitation, antibiotics for pPRoM, MgSO4 management of eclampsia, active management of the third stage of labor (AMTSL), and induction of labor for pregnancies lasting 41+ weeks. This can be modified by unchecking the box labeled "Allow LiST to calculate intervention coverages" on the "Childbirth" tab.

- **Default data source:** The percentage of deliveries at this level are calculated from Health Facility Deliveries (FacilDeliv) according to the following formula:
  -If FacilDeliv is less than 30%, then CEmOC deliveries are 10% of FacilDeliv
  -If FacilDeliv is between 30% and 50%, then CEmOC deliveries are 20% of FacilDeliv
  -If FacilDeliv is between 50% and 95%, then CEmOC deliveries are 60% of FacilDeliv - If FacilDeliv are 95% or greater, then CEmOC deliveries are 100% of FacilDeliv

- **Notes:**

Clean birth practices

- **Definition:** Percent of deliveries where clean birth practices are performed, including handwashing by the attendant, cleaning the maternal perineum, using a clean birth surface, clean cutting and tying of the cord, and hygienic cord and skin care immediately after delivery.

- **Default data source:** The default assumption is that 100% of SBAs in the home and 100% of institutional deliveries are using clean birth practices.

- **Notes:** The assumptions should be adjusted based on local knowledge and data. One potential choice is to use a clean delivery kit in the home for unassisted deliveries as an indicator of clean birth practices.

- **Effect size reference:** Note that the effectiveness of this intervention differs by level of delivery (see Effectiveness menu).

  Blencowe H, Cousens S, Mullany LC, et al. Clean birth and postnatal care practices to reduce neonatal deaths from sepsis and tetanus: A systematic review and Delphi estimation of mortality effect. BMC Public Health 2011; 11(Suppl 3): S11. http://www.ncbi.nlm.nih.gov/pmc/articles/PMC3231884/.

  Pollard SL, Mathai M, Walker N. Estimating the impact of interventions on cause-specific maternal mortality: A Delphi approach. BMC Public Health 2013; 13(Suppl 3): S12. http://www.ncbi.nlm.nih.gov/pmc/articles/PMC3847442/.

Immediate assessment and stimulation

- **Definition:** Percent of deliveries where rubbing and drying of the neonate immediately after delivery is performed.

- **Default data source:** The default assumption is that 100% of SBAs in the home and 100% of institutional deliveries are using appropriate and immediate assessment and stimulation.

- **Notes:**

- **Effect size reference:** Note that it is possible to vary the effectiveness of this intervention by level of delivery (see Effectiveness menu).

  Lee AC, Cousens S, Mullany LC, et al. Neonatal resuscitation and immediate newborn assessment and stimulation for the prevention of neonatal deaths: A systematic review, meta-analysis and Delphi estimation of mortality effect. BMC Public Health 2011; 11(Suppl 3): S12.

  http://www.ncbi.nlm.nih.gov/pubmed/21501429.

Labor and delivery management

- **Definition:** Percent of women receiving labor and delivery management from a skilled birth attendant.

- **Default data source:** The default assumption is that 100% of SBAs in the home and 100% of institutional deliveries have access to the appropriate facilities for the given level of care.

- **Notes:** The default assumption is that 100% of SBAs in the home and 100% of facility deliveries are able to provide labor and delivery management. However, the nature of this labor and delivery management is assumed to differ based on the location at which the delivery occurs (because of the differing availability of supplies, equipment, and skills of birth attendants at different levels of care). For this reason, the intervention of "labor and delivery management" has different effectiveness values at different levels of care.

- **Effect size reference:** Note that the effectiveness of this intervention differs by level of delivery (see Effectiveness menu).

  Lee AC, Cousens S, Darmstadt GL, et al. Care during labor and birth for the prevention of intrapartumrelated neonatal deaths: A systematic review and Delphi estimation of mortality effect. BMC Public Health 2011; 11(Suppl 3): S10. http://www.ncbi.nlm.nih.gov/pubmed/21501427.

  Yakoob MY, Ali MA, Ali MU, et al. The effect of providing skilled birth attendance and emergency obstetric care in preventing stillbirths. BMC Public Health 2011; 11(Suppl 3): S7. http://www.ncbi.nlm.nih.gov/pubmed/21501458.

  (Maternal mortality effect size from unpublished calculations based on: Pollard SL, Mathai M, Walker N.
  Estimating the impact of interventions on cause-specific maternal mortality: A Delphi approach. BMC Public Health 2013; 13(Suppl 3): S12. http://www.ncbi.nlm.nih.gov/pmc/articles/PMC3847442/.)

Neonatal resuscitation

- **Definition:** Percent of deliveries with access to detection of breathing problems and resuscitation (with a mucous extractor).

- **Default data source:** The default assumption is that 100% of institutional deliveries have access to neonatal resuscitation, if needed.

- **Notes:** We do not assume that neonatal resuscitation is performed in the home.

- **Effect size reference:** Note that the effectiveness of this intervention differs by level of delivery (see Effectiveness menu).

  Lee AC, Cousens S, Mullany LC, et al. Neonatal resuscitation and immediate newborn assessment and stimulation for the prevention of neonatal deaths: A systematic review, meta-analysis and Delphi estimation of mortality effect. BMC Public Health 2011; 11(Suppl 3): S12. http://www.ncbi.nlm.nih.gov/pubmed/21501429.

Antenatal corticosteroids for pre-term labor

- **Definition:** Percent of women with premature labor receiving an intramuscular injection of betamethasone sodium phosphate (6mg, every 12 hours for 2 days).

- **Default data source:** The default assumption is that 0% of births receive antenatal corticosteroids; user should enter local data if possible and available.

- **Notes:** Antenatal corticosteroids (ACS) were previously included in LiST as an intervention that could be effective in reducing deaths due to prematurity in 2006, based on work for the 2005 Lancet Neonatal series. (Darmstadt GL, Bhutta ZA, Cousens S, et al. Evidence-based, cost-effective interventions: how many newborns can we save and at what cost? Lancet 2005; 365: 988-97. http://www.ncbi.nlm.nih.gov/pubmed/15767001.) (Mwansa-Kambafwile J, Cousens S, Hanset T, et al. Antenatal steroids in preterm labour for the prevention of neonatal deaths due to complications of preterm birth. International Journal of Epidemiology 2010; 39(Suppl 1): i122-i133. http://www.ncbi.nlm.nih.gov/pmc/articles/PMC2845868/.) Since that time, there has been an increasing amount of information that finds that there can be negative consequences to the use of ACS, especially when provided in weaker health care systems. (Althabe F, Belizan JM, McClure EM, et al. A populationbased, multifaceted strategy to implement antenatal corticosteroid treatment versus standard care for the reduction of neonatal mortality due to preterm birth in low-income and middle-income countries: the ACT cluster-randomised trial. Lancet 2015; 385:629-39. http://www.ncbi.nlm.nih.gov/pubmed/25458726.) (Azad A, Costello A. Extreme caution is needed before scale-up of antenatal corticosteroids to reduce preterm deaths in low-income settings. Lancet Global Health 2014; 2: e191-2. http://www.sciencedirect.com/science/article/pii/S2214109X14700208.) We have decided that the best way to ensure that users do not miss-apply the possible impact of ACS on neonatal mortality is to leave the intervention in LiST, but to change the default effectiveness of this intervention to zero. This way if users to do want to model the impact of ACS when provided effectively in a stronger health care system they can do so, but will need to specify the effectiveness of the intervention.

- **Effect size reference:** The effect size is currently set to 0 (see note above); use the "Effectiveness" menu to modify if necessary.

Antibiotics for pPRoM

- **Definition:** Percent of pregnant women with premature rupture of the membranes (pPRoM) who are not in labor and are given oral erythromycin (250mg, 4 times daily for 7 days) to prevent infection.

- **Default data source:** The default assumption is that 100% of BEmOC and 100% of CEmOC deliveries have access to antibiotics for preterm prelabor rupture of membranes, if needed.

- **Notes:**

- **Effect size reference:** Note that it is possible to vary the effectiveness of this intervention by level of delivery (see Effectiveness menu).

  Cousens S, Blencowe H, Gravett M, et al. Antibiotics for pre-term pre-labour rupture of the membranes: Prevention of neonatal deaths due to complications of preterm birth and infection. International Journal of Epidemiology 2010; 39(Suppl 1): i34-i43. http://www.ncbi.nlm.nih.gov/pubmed/20348116.

$MgSO_4$ - management of eclampsia

- **Definition:** Percent of pregnant women receiving magnesium sulfate for eclampsia during delivery.

- **Default data source:** The default assumption is that 100% of BEmOC and 100% of CEmOC deliveries have access to $MgSO_4$ for eclampsia, if needed.

- **Notes:**

- **Effect size reference:** Note that it is possible to vary the effectiveness of this intervention by level of delivery (see Effectiveness menu).

  Ronsmans C, Campbell O. Quantifying the fall in mortality associated with interventions related to hypertensive diseases of pregnancy. BMC Public Health 2011; 11(Suppl 3): S8. http://www.ncbi.nlm.nih.gov/pubmed/21501459.

  Jabeen M, Yakoob MY, Imdad A, et al. Impact of interventions to prevent and manage preeclampsia and eclampsia on stillbirths. BMC Public Health 2011; 11(Suppl 3): S6. http://www.ncbi.nlm.nih.gov/pubmed/21501457.

AMTSL - Active Management of the Third Stage of Labor

- **Definition:** Percent of women with their third stage of labor managed actively. Active management of the third stage of labor (AMTSL) is defined as controlled cord traction, uterine massage, and appropriate oxytocics.

- **Default data source:** The default assumption is that 100% of BEmOC and 100% of CEmOC deliveries have appropriate active management during and after delivery.

- **Notes:** Note that it is possible to vary the effectiveness of this intervention by level of delivery (see Effectiveness menu).

  Pollard SL, Mathai M, Walker N. Estimating the impact of interventions on cause-specific maternal mortality:
  A Delphi approach. BMC Public Health 2013; 13(Suppl 3): S12.
  http://www.ncbi.nlm.nih.gov/pmc/articles/PMC3847442/.

Induction of labor for pregnancies lasting 41+ weeks

- **Definition:** Percent of women who are 41 or more weeks pregnant who are managed with induction of labor as appropriate.
- **Default data source:** The default assumptions are that 100% of CEmOC deliveries have access to induction of labor for post-term pregnancies, if needed.
- **Notes:** The current impact of this intervention is only on stillbirths.
- **Effect size reference:** Note that it is possible to vary the effectiveness of this intervention by level of delivery (see Effectiveness menu).

Hussain AA, Yakoob MY, Imdad A, et al. Elective induction for pregnancies at or beyond 41 weeks of gestation and its impact on stillbirths: A systematic review with meta-analysis. BMC Public Health 2011; 11 (Suppl 3): S5. http://www.ncbi.nlm.nih.gov/pubmed/21501456.

Breastfeeding

Exclusive breastfeeding Predominant breastfeeding Partial breastfeeding Any breastfeeding No breastfeeding Early initiation of breastfeeding Breastfeeding promotion

In the "Breastfeeding" tab, data may be entered by either **breastfeeding prevalence** or **breastfeeding promotion**. If prevalence is selected (default), the rates of exclusive, predominant, and partial breastfeeding may be modified. If promotion is selected, the coverage of breastfeeding promotion as an intervention may be modified and breastfeeding rates are automatically calculated as a result.

Exclusive breastfeeding

- **Definition:** Percent of children receiving only breastmilk for food (plus medication, vaccines, and vitamins).
- **Default data source:** Coverage data for this indicator are drawn from DHS, MICS, and other nationally representative household surveys; however, they have been recalculated from the raw data sources to reflect the entire age period of interest.
- **Notes:** This applies to children 0-1 months and 1-5 months of age.

  If recalculated data are not available, 0-2 months can be used as a proxy for 0-1 months while 4-5 months can be used as a proxy for 1-5 months.
- **Effect size references:** Lamberti LM, Zakarija-Grković I, Fischer Walker CL, et al. Breastfeeding for reducing the risk of pneumonia morbidity and mortality in children under two: A systematic literature review and meta-analysis. BMC Public Health 2013; 13(Suppl 3): S18. http://www.ncbi.nlm.nih.gov/pubmed/24564728.

  Black RE, Victora CG, Walker SP, et al. Maternal and child undernutrition and overweight in low-income and middle-income countries. Lancet 2013; 382(9890): 427-51. http://www.ncbi.nlm.nih.gov/pubmed/23746772.

  LiST Technical Note: Breastfeeding effect sizes on mortality in LiST.

http://livessavedtool.org/images/documents/Technical_Notes/Breastfeeding-effect-sizes-on-mortality.pdf

Predominant breastfeeding

- **Definition:** Percent of children receiving only breastmilk plus water and/or other non-milk liquids such as juices (plus medication, vaccines, and vitamins).

- **Default data source:** Coverage data for this indicator are drawn from DHS, MICS, and other nationally representative household surveys; however, they have been recalculated from the raw data sources to reflect the entire age period of interest.

- **Notes:** This applies to children 0-1 months and 1-5 months of age.

  If recalculated data are not available, 0-2 months has been used as a proxy for 0-1 months while 4-5 months has been used as a proxy for 1-5 months.

- **Effect size references:** Lamberti LM, Zakarija-Grković I, Fischer Walker CL, et al. Breastfeeding for reducing the risk of pneumonia morbidity and mortality in children under two: A systematic literature review and meta-analysis. BMC Public Health 2013; 13(Suppl 3): S18. http://www.ncbi.nlm.nih.gov/pubmed/24564728.

  Black RE, Victora CG, Walker SP, et al. Maternal and child undernutrition and overweight in low-income and middle-income countries. Lancet 2013; 382(9890): 427-51. http://www.ncbi.nlm.nih.gov/pubmed/23746772.

  LiST Technical Note: Breastfeeding effect sizes on mortality in LiST. http://livessavedtool.org/images/documents/Technical_Notes/Breastfeeding-effect-sizes-on-mortality.pdf

Partial breastfeeding

- **Definition:** Percent of children receiving breastmilk plus complementary foods and/or milk-based liquids (plus medication, vaccines, and vitamins).

- **Default data source:** Coverage data for this indicator are drawn from DHS, MICS, and other nationally representative household surveys; however, they have been recalculated from the raw data sources to reflect the exact age period of interest.

- **Notes:** This applies to children 0-1 months and 1-5 months of age.

  If recalculated data are not available, 0-2 months has been used as a proxy for 0-1 months while 4-5 months has been used as a proxy for 1-5 months.

- **Effect size references:** Lamberti LM, Zakarija-Grković I, Fischer Walker CL, et al. Breastfeeding for reducing the risk of pneumonia morbidity and mortality in children under two: A systematic literature review and meta-analysis. BMC Public Health 2013; 13(Suppl 3): S18. http://www.ncbi.nlm.nih.gov/pubmed/24564728.

  Black RE, Victora CG, Walker SP, et al. Maternal and child undernutrition and overweight in low-income and middle-income countries. Lancet 2013; 382(9890): 427-51.

http://www.ncbi.nlm.nih.gov/pubmed/23746772.

LiST Technical Note: Breastfeeding effect sizes on mortality in LiST. http://livessavedtool.org/images/documents/Technical_Notes/Breastfeeding-effect-sizes-on-mortality.pdf

Any breastfeeding

- **Definition:** The percent of children still receiving any breastmilk.

- **Default data source:** Coverage data for this indicator are drawn from DHS, MICS, and other nationally representative household surveys; however, they have been recalculated from the raw data sources to reflect the exact age period of interest.

- **Notes:** This applies to children 6-11 months and 12-23 months of age. No health benefit is assumed to accrue to children breastfed after 24 months of age.

- **Effect size references:** Lamberti LM, Zakarija-Grković I, Fischer Walker CL, et al. Breastfeeding for reducing the risk of pneumonia morbidity and mortality in children under two: A systematic literature review and meta-analysis. BMC Public Health 2013; 13(Suppl 3): S18. http://www.ncbi.nlm.nih.gov/pubmed/24564728.

  Black RE, Victora CG, Walker SP, et al. Maternal and child undernutrition and overweight in low-income and middle-income countries. Lancet 2013; 382(9890): 427-51. http://www.ncbi.nlm.nih.gov/pubmed/23746772.

  LiST Technical Note: Breastfeeding effect sizes on mortality in LiST. http://livessavedtool.org/images/documents/Technical_Notes/Breastfeeding-effect-sizes-on-mortality.pdf

No breastfeeding

- **Definition:** The percent of children not receiving any breastmilk.

- **Default data source:** Coverage data for this indicator are drawn from DHS, MICS, and other nationally representative household surveys.

- **Notes:** This can apply to children of any age group 0-23 months. It is calculated automatically as 100% minus the percentage of children in that age group with any level of breastfeeding.

- **Effect size references:** Lamberti LM, Zakarija-Grković I, Fischer Walker CL, et al. Breastfeeding for reducing the risk of pneumonia morbidity and mortality in children under two: A systematic literature review and meta-analysis. BMC Public Health 2013; 13(Suppl 3): S18. http://www.ncbi.nlm.nih.gov/pubmed/24564728.

  Black RE, Victora CG, Walker SP, et al. Maternal and child undernutrition and overweight in low-income and middle-income countries. Lancet 2013; 382(9890): 427-51. http://www.ncbi.nlm.nih.gov/pubmed/23746772.

  LiST Technical Note: Breastfeeding effect sizes on mortality in LiST.

http://livessavedtool.org/images/documents/Technical_Notes/Breastfeeding-effect-sizes-on-mortality.pdf

Early initiation of breastfeeding

- **Definition:** The percent of children who begin breastfeeding within 1 hour of birth.
- **Default data source:** Coverage data for this indicator are drawn from DHS, MICS, and other nationally representative household surveys.
- **Notes:**
- **Effect size references:** NEOVITA Study Group. Timing of initiation, patterns of breastfeeding, and infant survival: prospective analysis of pooled data from three randomised trials. Lancet Global Health 2016; 4 (4): e266-75. https://www.ncbi.nlm.nih.gov/pubmed/27013313.

  LiST Technical Note: Breastfeeding effect sizes on mortality in LiST. http://livessavedtool.org/images/documents/Technical_Notes/Breastfeeding-effect-sizes-on-mortality.pdf

Breastfeeding promotion

- **Definition:** Percentage of children whose mothers receive activities designed to promote breastfeeding. Breastfeeding promotion can either be one-on-one or group meetings. Promotion activities can take place within the health system, at the home/community level, or both.

  Health system interventions include the Baby-Friendly Hospital Initiative, establishment of rooming-in practices, organizational support on breastfeeding outcomes, etc.

  Home/community level interventions include one-on-one counseling by home visit or telephone, home support by father or grandparent, group counseling, group meetings, social mobilization, mass media, social media, etc.

- **Default data source:** Coverage data for this indicator are not typically available. As a proxy, the level of breastfeeding promotion is set by default to equal the percent of children 1-5 months of age that are exclusively breastfed; user should enter local data if possible and available.

Notes: It is assumed that children 1-5 months of age who are exclusively breastfed do not need this behavior.

Breastfeeding promotion is applied to breastfeeding prevalence as an increased odds of "age-appropriate breastfeeding" (that is, exclusive breastfeeding for children under 6 months, and any/continued breastfeeding for children 6-23 months). Each of the three promotion channels (health system, home/community, or both) has a different impact on breastfeeding prevalence.

- **Effect size reference:** Haroon S, Das JK, Salam RA, et al. Breastfeeding promotion interventions and breastfeeding practices: A systematic review. BMC Public Health 2013; 13(Suppl 3): S20. http://www.ncbi.nlm.nih.gov/pubmed/24564836.

  Bhutta ZA, Das JK, Rizvi A, et al. Evidence-based interventions for improvement of maternal and child nutrition: what can be done and at what cost? Lancet 2013; 382(9890): 352-77. http://www.ncbi.nlm.nih.gov/pubmed/23746776. (Supplemental material, page 16.)

  Sinha B, Chowdhury R, Sankar MJ, et al. Interventions to improve breastfeeding outcomes: A systematic review and meta-analysis. Acta Paediatrica 2015; 104(467): 114-34. http://www.ncbi.nlm.nih.gov/pubmed/26183031.

  Sinha B, Chowdhury R, Prakash Upadhyay R, Taneja S, Martines J, Bahl R, Jeeva Sankar M; Integrated
  Interventions Delivered in Health Systems, Home, and Community Have the Highest Impact on Breastfeeding Outcomes in Low- and Middle-Income Countries. Journal Nutr 2017. 147(11S): 2179S– 2187S, https://doi.org/10.3945/jn.116.242321.

Stunting

When the "direct entry of stunting" option is checked in the LiST configuration menu, the stunting tab appears in the Coverage menu. Within this tab, users may modify stunting rates either through the "single indicator for stunting" option or the "detailed indicators for stunting" option.

The purpose of the single indicator is aggregate the bottom (worst) two stunting categories to create a single percentage of children who are stunted. (For example, if 2% of children are in the <-3 Z-score category and 5% of children are in the -3 to -2 z-score category, the single indicator will display "7% of children stunted.") It also aggregates across age groups, weighting by the number of months in each age range (i.e., <1 month is assumed to represent 1/60 of children, 1-5 months is assumed to represent 5/60 of children, etc.).

When the "single indicator" option is selected, the first (baseline) year of the single indicator will be grayed out and the rest of the years will be active. In the detailed display, the first year will be active (allowing users to edit baseline data here if necessary), but the following years will be grayed out. When the "detailed display" option is selected, the single indicator will remain visible but will be entirely grayed out, and the detailed display will be fully active.

Please note that in a healthy population (assumed to have a normal distribution of height for age), approximately 2.275% of the population will fall below -2 z-scores (i.e., more than 2 standard deviations below the median). As a result, we do not recommend setting the single indicator for stunting at a rate below 2.275%, as this is below what is considered normal for a healthy population.

Stunting distributions

- **Definition:** Distribution of the percent of children falling into one of four Z-score categories for height for age: <-3Z (severe stunting), -3 to -2Z (moderate stunting), -2 to -1Z (mild stunting), and >-1Z (not stunted). Stunting reflects chronic undernutrition.

- **Default data source:** Data have been recalculated for consistency across countries, using DHS and MICS datasets. This also ensures that the values reflect the exact age groups of interest.

- **Notes:** Disaggregated data are not available for 0-1 and 1-5 months. As a proxy, the 0-6 month values are applied to both age groups.

Preventive

Clean postnatal care practices Chlorhexidine Complementary feeding - education only Complementary feeding - education and supplementation Vitamin A supplementation Zinc supplementation Improved water source Water connection in the home Improved sanitation Hand washing with soap Hygienic disposal of children's stools ITN/IRS

Clean postnatal care practices

- **Definition:** Percent of neonates where the mother washes her hands frequently, the child lives in a clean environment, and no harmful practices are performed.

- **Default data source:** Coverage data for this indicator are not typically available. As a proxy, it is assumed that all neonates receiving a preventive postnatal visit within 48 hours of delivery will subsequently receive adequate clean postnatal practices in the home. Coverage data for this proxy indicator are drawn from DHS, MICS, and other nationally representative household surveys.

- **Notes:**

- **Effect size reference:** Blencowe H, Cousens S, Mullany LC, et al. Clean birth and postnatal care practices to reduce neonatal deaths from sepsis and tetanus: A systematic review and Delphi estimation of mortality effect. BMC Public Health 2011; 11(Suppl 3): S11. http://www.ncbi.nlm.nih.gov/pmc/articles/PMC3231884/.

Chlorhexidine

- **Definition:** Percent of neonates with chlorhexidine applied after birth to the cord.

- **Default data source:** Coverage data for this indicator are not typically available. Currently set at 0 for baseline; user should enter local data if possible and available.

- **Notes:**

- **Effect size reference:** Imdad A, Mullany LC, Baqui AH, et al. The effect of umbilical cord cleansing with chlorhexidine on omphalitis and neonatal mortality in community settings in developing countries: A meta -analysis. BMC Public Health 2013; 13(Suppl 3): S15. http://www.ncbi.nlm.nih.gov/pubmedhealth/PMH0061555/.

Complementary feeding - education only

- **Definition:** Percent of mothers intensively counseled on the importance of continued breastfeeding beyond six months and appropriate complementary feeding practices. As a proxy,

the percent of 6-23 month old children receiving minimum dietary diversity (4+ food groups) is used.

Default data source: Coverage data for this indicator are drawn from DHS, MICS, and other nationally representative household surveys.

- **Notes:** This is applied to the food secure population. The proxy used is those living on more than $1.90/day. This proxy can be modified in the Household Status tab of the "Health status, mortality, and economic status" menu.

  As a default, the two complementary feeding coverage indicators are equal (using the same proxy indicator of minimum dietary diversity). The user should enter local data to differentiate between the two indicators (education only vs. education and supplementation) if possible and available.

  The combined effect of both complementary feeding indicators will appear under Results as "appropriate complementary feeding."

- **Effect size reference:**

  Panjwani A and Heidkamp R. Complementary Feeding Interventions Have a Small but Significant Impact on Linear and Ponderal Growth of Children in Low- and Middle-Income Countries: A Systematic Review and Meta-Analysis. J Nutr 2017, 147(11S):2169S-2178S. https://doi.org/10.3945/jn.116.243857

  Imdad A, Yakoob MY, Bhutta ZA. Impact of maternal education about complementary feeding and provision of complementary foods on child growth in developing countries. BMC Public Health 2011; 11 (Suppl 3): S25. http://www.ncbi.nlm.nih.gov/pubmed/21501443.

  Bhutta ZA, Ahmed T, Black RE, et al. What works? Interventions for maternal and child undernutrition and survival. Lancet 2008; 371: 417-40. http://www.ncbi.nlm.nih.gov/pubmed/18206226.

Complementary feeding - education and supplementation

- **Definition:** Percent of mothers intensively counseled on the importance of continued breastfeeding beyond six months and appropriate complementary feeding practices, and given appropriate dietary supplementation. As a proxy, the percent of 6-23 month old children receiving minimum dietary diversity (4+ food groups) is used.

- **Default data source:** Coverage data for this indicator are drawn from DHS, MICS, and other nationally representative household surveys.

- **Notes:** This is applied to the food insecure population. The proxy used is those living on less than $1.90/day. This proxy can be modified in the Household Status tab of the "Health status, mortality, and economic status" menu.

  As a default, the two complementary feeding coverage indicators are equal (using the same proxy indicator of minimum dietary diversity). The user should enter local data to differentiate between the two indicators (education only vs. education and supplementation) if possible and available.

The combined effect of both complementary feeding indicators will appear under Results as "appropriate complementary feeding."

- **Effect size reference:** Imdad A, Yakoob MY, Bhutta ZA. Impact of maternal education about complementary feeding and provision of complementary foods on child growth in developing countries. BMC Public Health 2011; 11(Suppl 3): S25. http://www.ncbi.nlm.nih.gov/pubmed/21501443.

Bhutta ZA, Ahmed T, Black RE, et al. What works? Interventions for maternal and child undernutrition and survival. Lancet 2008; 371: 417-40. http://www.ncbi.nlm.nih.gov/pubmed/18206226.

Vitamin A supplementation

- **Definition:** Percent of children 6-59 months of age receiving two doses of Vitamin A during the last 12 months.
- **Default data source:** UNICEF - Vitamin A coverage. http://data.unicef.org/nutrition/vitamin-a. Updated annually.
- **Notes:** The full indicator is typically not available from a DHS/MICS or other household survey. However, the percent of children 6-59 months receiving 1 dose of Vitamin A in the past 6 months can be used if necessary from these sources.
- **Effect size reference:** Imdad A, Yakoob MY, Sudfeld CR, et al. Impact of vitamin A supplementation on infant and childhood mortality. BMC Public Health 2011; 11(Suppl 3): S20. http://www.ncbi.nlm.nih.gov/pubmed/21501438.

Zinc supplementation

- **Definition:** Percent of children 12-59 months of age who are given daily supplements of 10mg zinc.
- **Default data source:** Coverage data for this indicator are not typically available. Currently set at 0 for baseline; user should enter local data if possible and available.
- **Notes:** This is not the same indicator as zinc treatment for diarrhea.
- **Effect size reference:** Yakoob MY, Theodoratou E, Jabeen A, et al. Preventive zinc supplementation in developing countries: impact on mortality and morbidity due to diarrhea, pneumonia and malaria. BMC Public Health 2011; 11(Suppl 3): S23. http://www.ncbi.nlm.nih.gov/pubmed/21501441.

  Black RE, Victora CG, Walker SP, et al. Maternal and child undernutrition and overweight in low-income and middle-income countries. Lancet 2013; 382(9890): 427-51. http://www.ncbi.nlm.nih.gov/pubmed/23746772. (Supplemental material, page 18-19.)

Improved water source

- **Definition:** Percent of households with access to an improved water source within a 30 minute walk.

- **Default data source:** WHO/UNICEF Joint Monitoring Program (JMP) for Water Supply and Sanitation (http://www.wssinfo.org/). Data are available for all countries from 1996 to 2012.

- **Notes:** The effect of this intervention is applied to the difference between the percentage of households with access to an improved water source and the percentage of households with a connection in the home (which is a subset of improved water source). As a result, the value of this indicator must be greater than or equal to water connection in the home.

- **Effect size reference:** Cairncross S, Hunt C, Boisson S, et al. Water, sanitation and hygiene for the prevention of diarrhoea. International Journal of Epidemiology 2010; 39(Suppl 1): i193-i205. http://www.ncbi.nlm.nih.gov/pubmed/20348121.

Water connection in the home

Definition: Percent of households with a household connection, including water piped into the home or yard.

- **Default data source:** WHO/UNICEF Joint Monitoring Program (JMP) for Water Supply and Sanitation (http://www.wssinfo.org/). Data are available for all countries from 1996 to 2012.

- **Notes:** This is a subset of households with access to an improved water source. The model automatically ensures no double-counting of impact.

- **Effect size reference:** Cairncross S, Valdmanis V. Water supply, sanitation, and hygiene promotion. In:
  Jamison DT, Breman JG, Measham AR, et al., editors. Disease control priorities in developing countries. Washington DC: The World Bank, 2006; p. 771-792.
  http://www.ncbi.nlm.nih.gov/books/NBK11728/.

Improved sanitation

- **Definition:** Percent of households using an improved sanitation facility (defined as flush or pour flush to piped sewer system, septic tank, or pit latrine; ventilated improved pit (VIP) latrine; pit latrine with slab; or composting toilet).

- **Default data source:** WHO/UNICEF Joint Monitoring Program (JMP) for Water Supply and Sanitation (http://www.wssinfo.org/). Data are available for all countries from 1996 to 2012.

- **Notes:**

- **Effect size reference:** Cairncross S, Hunt C, Boisson S, et al. Water, sanitation and hygiene for the prevention of diarrhoea. International Journal of Epidemiology 2010; 39(Suppl 1): i193-i205. http://www.ncbi.nlm.nih.gov/pubmed/20348121. The effect is applied to the difference between an improved water source and a household connection.

Hand washing with soap

- **Definition:** Percent of mothers using appropriate handwashing practices, including washing hands with soap, ash, or other materials and using adequate water, after handling feces and before preparing food.

- **Default data source:** Curtis VA, Danguah LO, Aunger RV. Planned, motivated and habitual hygiene behaviour: An eleven country review. Health Education Research 2009; 24(4): 655-73. http://www.ncbi.nlm.nih.gov/pubmed/19286894. Table 3 of this paper provides data for a small set of countries (Ghana, Kenya, Kyrgyzstan, Madagascar, Peru, Senegal, Tanzania, and Uganda). All other countries are set by default to 17%, the global average cited in the paper.

- **Notes:** Neither reported handwashing behavior nor availability of handwashing materials in the home are appropriate indicators. Observational data are required.

- **Effect size reference:** Cairncross S, Hunt C, Boisson S, et al. Water, sanitation and hygiene for the prevention of diarrhoea. International Journal of Epidemiology 2010; 39(Suppl 1): i193-i205. http://www.ncbi.nlm.nih.gov/pubmed/20348121.

Hygienic disposal of children's stools

- **Definition:** Percent of children's stools that are disposed of safely and contained. Stools are considered to be contained if: 1) the child always uses a toilet/latrine, 2) the feces are thrown in the toilet/latrine, or 3) the feces are buried in the yard.

- **Default data source:** Coverage data for this indicator are drawn from DHS, MICS, and other nationally representative household surveys.

- **Notes:** In some countries, utilization of disposable diapers may be considered hygienic disposal, but this is included on a country-by-country basis as the DHS/MICS has chosen.

- **Effect size reference:** Presented at the 2008 CHERG meeting.

ITN/IRS - household ownership of insecticide treated bednet (ITN) and/or protected by indoor residual spraying (IRS)

- **Definition:** Percent of households owning at least one insecticide treated bednet (ITN) and/or protected by indoor residual spraying (IRS).

- **Default data source:** Coverage data for this indicator are drawn from DHS, MICS, and other nationally representative household surveys. This includes Malaria Indicator Surveys.

- **Notes:** For historical trends where data on IRS are not available, data on percent of households with one or more ITN are used instead; this is considered a reasonable minimum bound. Data points where this substitution is used are indicated in the source notes. ITNs are assumed to have been introduced in 2000, so a linear "scale-up" trend from zero in 1999 to the first available data point for the country is automatically computed.

  The indicator can be substituted with trends in "children sleeping under an ITN," but the estimate will be conservative. The default effect size is based on household ownership, not utilization. Note that any bednet (treated or untreated) is not an adequate indicator.

- **Effect size reference:** Eisele TP, Larsen D, Steketee RW. Protective efficacy of interventions for preventing malaria mortality in children in Plasmodium falciparum endemic areas. International Journal of Epidemiology 2010; 39(Suppl 1): i88-i101. https://www.ncbi.nlm.nih.gov/pmc/articles/PMC2845865/.

  Radeva-Petrova D, Kayentao K, Ter Kuile FO, et al. Drugs for preventing malaria in pregnant women in endemic areas: Any drug regimen versus placebo or no treatment. Cochrane Database Syst Rev 2014. http://www.ncbi.nlm.nih.gov/pmc/articles/PMC4498495/.

Vaccines

BCG Polio Pentavalent DPT Hib HepB Pneumococcal Rotavirus Measles Meningococcal A Diarrheal vaccine pathogen B Diarrheal vaccine pathogen C Malaria vaccine Vaccine D

Vaccines given to children in their first year of life have protective effects until they are five years of age. When editing vaccine coverage, users will have to specify coverage in the four years prior to the base year of coverage of the LiST module. This will allow the model to correctly compute the protective effects of vaccines for children in different age groups in a given year.

For each vaccine, the number of doses that is considered "full coverage" is displayed on the "Vaccines" tab. To enter coverage of incomplete doses and/or supplemental vaccination, double-click on the relevant vaccine. There are no default effectiveness estimates for supplemental vaccination (additional doses or "boosters"),

although there are effectiveness estimates for suboptimal dosing. The sum of all the doses must be less than or equal to 100% of children.

If a supplemental campaign is being modeled, check the box for "supplemental vaccination - campaign implemented" in the relevant year. Then enter data on geographic coverage, low and high age groups targeted, and the average number of doses per child. Select the radial button at the top of the page to enter either the number of doses provided or the percent of target population reached, and then enter those values at the bottom of the page. Default values are not available for this section of the model.

BCG

- **Definition:** Percent of live births receiving at least one dose of BCG.

- **Default data source:** Default data from WHO/UNICEF (http://apps.who.int/immunization_monitoring/globalsummary/timeseries/tswucoveragebcg.html), updated annually. These data can be replaced with DHS/MICS data if necessary.

- **Notes:** BCG is included in LiST to indicate that it is an important intervention delivered during the first five years of life.

- **Effect size reference:** The current assumption is that BCG has no measurable impact upon under-five mortality.

Polio

- **Definition:** Percent of children who survive the first year of life who have received at least three doses of polio vaccine.

- **Default data source:** Default data from WHO/UNICEF (http://apps.who.int/immunization_monitoring/globalsummary/timeseries/tswucoveragepol3.html), updated annually. These data can be replaced with DHS/MICS data if necessary.

- **Notes:** Polio vaccination is included in LiST to indicate that it is an important intervention delivered during the first five years of life.

- **Effect size reference:** The current assumption is that polio vaccination has no measurable impact upon under-five mortality.

Pentavalent

- **Definition:** Percent of children who survive the first year of life who have received 3 doses of pentavalent vaccine.

- **Default data source:** Default data from WHO/UNICEF (http://www.who.int/immunization/monitoring_surveillance/en/), updated annually. These data can be replaced with DHS/MICS data if necessary.

- **Notes:** Changing the pentavalent vaccine coverage will automatically change the coverage of DPT, Hib, and HepB. See the individual vaccines for effect information.

DPT

- **Definition:** Percent of children who survive the first year of life who have received 3 doses of DPT vaccine.

- **Default data source:** Default data from WHO/UNICEF (http://apps.who.int/immunization_monitoring/globalsummary/timeseries/tswucoveragedtp3.html), updated annually. These data can be replaced with DHS/MICS data if necessary.

- **Notes:** The effect of DPT is only on pertussis mortality. There is currently no impact on tetanus or diphtheria mortality. Default herd effect is 0. Note that herd immunity can be adjusted if desired.

- **Effect size reference:** Fulton TR, Phadke VK, Orenstein WA, et al. Protective Effect of Contemporary Pertussis Vaccines: A Systematic Review and Meta-analysis. Clinical Infectious Diseases 2016; 62(9): 1100 -1100. http://www.ncbi.nlm.nih.gov/pubmed/26908803.

Hib

- **Definition:** Percent of children who survive the first year of life who have received 3 doses of Hib vaccine.

- **Default data source:** Default data from WHO/UNICEF (http://apps.who.int/immunization_monitoring/globalsummary/timeseries/tswucoveragehib3.html), updated annually. These data can be replaced with DHS/MICS data if necessary.

- **Notes:** Default herd effect is 0. Note that herd immunity can be adjusted.

- **Effect size reference:** Griffiths UK, Clark A, Gessner B, et al. Dose-specific efficacy of Haemophilus influenzae type b conjugate vaccines: A systematic review and meta-analysis of controlled clinical trials.
  Epidemiology and Infection 2012; 140(8): 1343-1355.
  http://www.ncbi.nlm.nih.gov/pubmed/22583474.

HepB

- **Definition:** Percent of live births who have received at least three doses of Hepatitis B vaccine.

- **Default data source:** Default data from WHO/UNICEF (http://apps.who.int/immunization_monitoring/globalsummary/timeseries/tswucoveragehepb3.html), updated annually. These data can be replaced with DHS/MICS data if necessary.

- **Notes:** Hepatitis B vaccination is included in LiST to indicate that it is an important intervention delivered during the first five years of life.

- **Effect size reference:** The current assumption is that hepatitis B vaccination has no measurable impact upon under-five mortality.

Pneumococcal

- **Definition:** Percent of children who survive the first year of life who have received 3 doses of Pneumococcal vaccine.

- **Default data source:** Default data from WHO/UNICEF (http://apps.who.int/immunization_monitoring/globalsummary/timeseries/tswucoveragepcv3.html), updated annually. These data can be replaced with DHS/MICS data if necessary.

- **Notes:** Default herd effect is 0. Note that herd immunity can be adjusted.

- **Effect size reference:** Lucero MG, Dulalia VE, Nillos LT, et al. Pneumococcal conjugate vaccines for preventing vaccine-type invasive pneumococcal disease and pneumonia with consolidation on x-ray in children under two years of age. Cochrane Database Syst Rev 2009. http://www.ncbi.nlm.nih.gov/pubmed/19821336.

Rotavirus

- **Definition:** Percent of children who survive the first year of life who have received 2 or 3 doses of Rotavirus vaccine (according to manufacturer's schedule).

- **Default data source:** Default data from WHO/UNICEF (http://apps.who.int/immunization_monitoring/globalsummary/timeseries/tswucoveragerota_last.html), updated annually. These data can be replaced with DHS/MICS data if necessary.

- **Notes:** The effect size varies by geographic region. Default herd effect is 0. Note that herd immunity can be adjusted.

- **Effect size reference:** Fisher Walker CL, Black RE. Rotavirus vaccine and diarrhea mortality: Quantifying regional variation in effect size. BMC Public Health 2011; 11(Suppl 3): S16. http://www.ncbi.nlm.nih.gov/pmc/articles/PMC3231889/.

Measles

- **Definition:** Percent of children who survive the first year of life who have received 1 dose of measles vaccine.

- **Default data source:** Default data from WHO/UNICEF (http://apps.who.int/immunization_monitoring/globalsummary/timeseries/tswucoveragemcv.html), updated annually. These data can be replaced with DHS/MICS data if necessary.

- **Notes:** The default model is appropriate for vaccines which have already been rolled out, but not necessarily for new vaccines. The model may not necessarily correctly estimate the time to elimination of disease (at the very extremes of coverage) but will correctly model control of disease. Note that the impact of herd immunity can be adjusted. Default herd effect is 100% at 95% coverage. The effect of supplemental campaigns and second-opportunity measles vaccination can also be calculated.

- **Effect size reference:** Sudfeld CR, Navar AM, Halsey NA. Effectiveness of measles vaccination and vitamin A treatment. International Journal of Epidemiology 2010; 39(Suppl 1): i48-i55. http://www.ncbi.nlm.nih.gov/pubmed/20348126.

Meningococcal A

- **Definition:** Percent of children who survive the first year of life who have received a full course of Meningococcal A vaccine (according to manufacturer's schedule).

- **Default data source:** There is no data currently available.

- **Notes:** For default, we assume a single dose is full coverage. This is a placeholder for research purposes. There is no default effect size. To use this indicator, the user must enter their predicted data. This is indicator is not available in the standard model.

- **Effect size reference:** None.

Diarrheal Vaccine Pathogen B

- **Definition:** Percent of children children who survive the first year of life who have received a full course of Pathogen B vaccine.

- **Default data source:** N/A

- **Notes:** For default, we assume a single dose is full coverage. This is a placeholder for research purposes. There is no default effect size. To use this indicator, the user must enter data. This indicator is not available in the standard model.

- **Effect size reference:** None.

Diarrheal Vaccine Pathogen C

- **Definition:** Percent of children children who survive the first year of life who have received a full course of Pathogen C vaccine.

- **Default data source:** N/A

- **Notes:** For default, we assume a single dose is full coverage. This is a placeholder for research purposes. There is no default effect size. To use this indicator, the user must enter data. This indicator is not available in the standard model.

- **Effect size reference:** None.

Malaria Vaccine

- **Definition:** Percent of children who survive the first year of life who have received a full course of malaria vaccine.

- **Default data source:** N/A

- **Notes:** For default, we assume a single dose is full coverage. This is a placeholder for research purposes. There is no default effect size. To use this indicator, the user must enter data. This is indicator is not available in the standard model.

- **Effect size reference:** None.

Vaccine D

- **Definition:** Percent of children who survive the first year of life who have received a full course of vaccine D.

- **Default data source:** N/A

- **Notes:** For default, we assume a single dose is full coverage. This is a placeholder for research purposes. There is no default effect size. To use this indicator, the user must enter data. This is indicator is not available in the standard model.

- **Effect size reference:** None.

Curative

Maternal sepsis case management Case management of premature babies Thermal care KMC - Kangaroo Mother Care Full supportive care for premature babies Case management of severe neonatal infections Oral antibiotics Injectable antibiotics Full supportive care for sepsis/pneumonia ORS Antibiotics - treatment for dysentery Zinc - treatment of diarrhea Oral antibiotics for pneumonia Vitamin A - treatment of measles Antimalarials - Artemesinin compounds for malaria Therapeutic feeding for severe wasting Treatment for moderate acute malnutrition Cotrimoxazole ART (for children)

Maternal sepsis case management

- **Definition:** Percent of newly delivered mothers with suspected sepsis managed at a Basic Emergency Obstetric Care (BEmOC) level.

- **Default data source:** Coverage data for this indicator are not typically available. Currently set at 0 for baseline; user should enter local data if possible and available.

- **Notes:**

- **Effect size reference:** Pollard SL, Mathai M, Walker N. Estimating the impact of interventions on causespecific maternal mortality: A Delphi approach. BMC Public Health 2013; 13(Suppl 3): S12. http://www.ncbi.nlm.nih.gov/pmc/articles/PMC3847442/.

Case management of premature babies

- **Definition:** This refers to the sum of the three levels of management of prematurely born infants in the neonatal period: thermal care, Kangaroo mother care, and full supportive care for prematurity. See below for details on each intervention.

- **Default data source:** N/A

- **Notes:** The sum of the coverage values for the three levels of care must be less than or equal to 100%.

- **Effect size reference:** This intervention does not have a direct effect, but refers to the sum of the three indicators listed below.

Thermal care

Definition: Percent of neonates whose mother delays the infant's bath and practices skin-to-skin contact in order to maintain thermal control of the infant.

- **Default data source:** Coverage data for this indicator are not typically available. As a proxy, it is assumed that all babies delivered in a health facility will receive appropriate thermal care. (See pregnancy intervention coverage for further information on delivery locations.)
- **Notes:** This only benefits premature infants.
- **Effect size reference:** Bhutta ZA, Das JK, Bahl R, et al. Can available interventions end preventable deaths in mothers, newborn babies, and stillbirths, and at what cost? Lancet 2014; 384(9940): 347-70. http://www.ncbi.nlm.nih.gov/pubmed/24853604. (Supplemental material, page 33.)

KMC - Kangaroo Mother Care

- **Definition:** Percent of premature neonates receiving facility-based Kangaroo Mother Care (KMC). KMC is defined as continuous skin-to-skin contact between a mother and her newborn as well as frequent and exclusive breastfeeding.
- **Default data source:** Coverage data for this indicator are not typically available. Currently set at 0 for baseline; user should enter local data if possible and available.
- **Notes:** This only benefits premature infants. The affected fraction is the percent of premature births that survive to day 3 (an average of 58%).

  There is inadequate data to include community-based KMC.

  KMC has an effect in LiST on rates of exclusive breastfeeding (through the breastfeeding promotion component of KMC). This will appear in LiST results under "changes in breastfeeding" (rather than being attributed directly to KMC).
- **Effect size reference:** Lawn JE, Mwansa-Kambafwile J, Horta BL, et al. 'Kangaroo Mother Care' to prevent deaths due to preterm birth complications. International Journal of Epidemiology 2010; 39(Suppl 1): i44-i54. http://www.ncbi.nlm.nih.gov/pubmed/20348117.

  Boundy EO, Dastjerdi R, Spiegelman D, et al. Kangaroo mother care and neonatal outcomes: A metaanalysis. Pediatrics 2016; 137(1). http://www.ncbi.nlm.nih.gov/pubmed/26702029.

Full supportive care for premature babies

- **Definition:** Percent of prematurely born neonates who have access to and receive hospital-based full supportive care, including KMC, feeding support/IV fluids, infection prevention/management, oxygen provision, management of neonatal jaundice, nasal CPAP/IPPV (as required), and surfactant for Respiratory Distress Syndrome.

- **Default data source:** Coverage data for this indicator are not typically available. Currently set at 0 for baseline; user should enter local data if possible and available.

- **Notes:**

- **Effect size reference:** Bhutta ZA, Das JK, Bahl R, et al. Can available interventions end preventable deaths in mothers, newborn babies, and stillbirths, and at what cost? Lancet 2014; 384(9940: 347-70. http://www.ncbi.nlm.nih.gov/pubmed/24853604.

Case management of severe neonatal infection

- **Definition:** This refers to the sum of the three levels of case management for severe infection in the neonatal period: oral antibiotics, injectable antibiotics, and full supportive care. See below for details on each intervention.

- **Default data source:** N/A

- **Notes:** The sum of the three levels of care must be less than or equal to 100%.

- **Effect size reference:** This intervention does not have a direct effect, but refers to the sum of the three indicators listed below.

Oral antibiotics

- **Definition:** Percent of neonates with suspected sepsis/pneumonia treated with oral antibiotics.

- **Default data source:** Coverage data for this indicator are not typically available. Currently set at 0 for baseline; user should enter local data if possible and available.

- **Notes:**

- **Effect size reference:** Zaidi AK, Ganatra HA, Syed S, et al. Effect of case management on neonatal mortality due to sepsis and pneumonia. BMC Public Health 2011; 11(Suppl 3): S13. http://www.ncbi.nlm.nih.gov/pubmed/21501430.

Injectable antibiotics

- **Definition:** Percent of neonates with suspected sepsis/pneumonia treated with injectable antibiotics.

- **Default data source:** Coverage data for this indicator are not typically available. As a proxy, it is assumed that all babies delivered in a health facility will receive injectable antibiotics if needed. (See pregnancy intervention coverage for further information on delivery locations.)

- **Notes:**

- **Effect size reference:** Zaidi AK, Ganatra HA, Syed S, et al. Effect of case management on neonatal mortality due to sepsis and pneumonia. BMC Public Health 2011; 11(Suppl 3): S13.

http://www.ncbi.nlm.nih.gov/pubmed/21501430.

Full supportive care for sepsis/pneumonia

- **Definition:** Percent of neonates with suspected sepsis/pneumonia treated with hospital-based full supportive care, including oxygen, IV fluids, IV antibiotics, blood transfusion, phototherapy, etc. as needed.
- **Default data source:** Coverage data for this indicator are not typically available. Currently set at 0 for baseline; user should enter local data if possible and available.
- **Notes:**
- **Effect size reference:** Zaidi AK, Ganatra HA, Syed S, et al. Effect of case management on neonatal mortality due to sepsis and pneumonia. BMC Public Health 2011; 11(Suppl 3): S13. http://www.ncbi.nlm.nih.gov/pubmed/21501430.

ORS - oral rehydration solution

- **Definition:** Percent of children 0-59 months with suspected diarrhea treated with oral rehydration solution (ORS), including sachets or pre-mixed solutions. This indicator does not include homemade sugar -salt solution or recommended home fluids due to lack of adequate data.
- **Default data source:** Coverage data for this indicator are drawn from DHS, MICS, and other nationally representative household surveys.
- **Notes:** It is assumed that 88% of diarrhea mortality is susceptible to treatment with ORS. (Source: Rahman AE, Moinuddin M, Molla M, et al. Childhood diarrhoeal deaths in seven low- and middle-income countries. Bulletin WHO 2014. https://www.ncbi.nlm.nih.gov/pubmed/25378757.) To change this assumption, visit the Effectiveness menu to alter the affected fraction.

  This is an indicator of appropriate diarrhea treatment. This does not suggest that increased fluids, continuous feeding, or ORT should not be recommended.
- **Effect size reference:** Munos M, Fischer Walker CL, Black RE. The effect of oral rehydration solution and recommended home fluids on diarrhea mortality. International Journal of Epidemiology 2010; 39 (Suppl 1): i75-i87. http://www.ncbi.nlm.nih.gov/pubmed/20348131.

Antibiotics - treatment for dysentery

- **Definition:** Percent of children 0-59 months with bloody diarrhea who receive appropriate antibiotic treatment (including ciprofloxacin, ceftriaxone, and pivmecillinam).
- **Default data source:** Coverage data for this indicator are drawn from DHS, MICS, and other nationally representative household surveys.
- **Notes:** It is assumed that 12% of diarrhea mortality is due to dysentery, and hence susceptible to treatment with this intervention. (Source: Rahman AE, Moinuddin M, Molla M, et al. Childhood diarrhoeal deaths in seven low- and middle-income countries. Bulletin WHO 2014.

https://www.ncbi.nlm.nih.gov/pubmed/25378757.) To change this assumption, visit the Effectiveness menu to alter the affected fraction.

- **Effect size reference:** Das JK Ali A, Salam RA, et al. Antibiotics for the treatment of Cholera, Shigella and Cryptosporidium in children. BMC Public Health 2013; 13(Suppl 3): S10. http://www.ncbi.nlm.nih.gov/pubmed/24564492.

Zinc - treatment of diarrhea

- **Definition:** Percent of children 0-59 months with suspected diarrhea treated with 20mg of zinc daily.
- **Default data source:** Coverage data for this indicator are drawn from DHS, MICS, and other nationally representative household surveys.
- **Notes:**
- **Effect size reference:** Fischer Walker CL, Black RE. Zinc for the treatment of diarrhea: Effect on diarrhea morbidity, mortality and incidence of future episodes. International Journal of Epidemiology 2010; 39(Suppl 1): i63-i69. http://www.ncbi.nlm.nih.gov/pubmed/20348128.

Oral antibiotics for pneumonia

- **Definition:** Percent of children with suspected pneumonia (symptoms of acute respiratory infection) for whom advice or treatment was sought from a health facility or provider.
- **Default data source:** Coverage data for this indicator are drawn from DHS, MICS, and other nationally representative household surveys. Careseeking for pneumonia is used as a proxy for treatment with oral antibiotics.
- **Notes:**
- **Effect size reference:** Theodoratou E, Al-Jilaihawi S, Woodward F, et al. The effect of case management on childhood pneumonia mortality in developing countries. International Journal of Epidemiology 2010; 39(Suppl 1): i155-i171. http://www.ncbi.nlm.nih.gov/pubmed/20348118.

Vitamin A - treatment of measles

- **Definition:** Percent of children with measles treated with Vitamin A.
- **Default data source:** Coverage data for this indicator are not typically available. As a proxy, the percent of children 6-59 months receiving two doses of vitamin A in 12 months (preventive Vitamin A supplementation) is used.
- **Notes:**
- **Effect size reference:** Sudfeld CR, Navar AM, Halsey NA. Effectiveness of measles vaccination and vitamin A treatment. International Journal of Epidemiology 2010; 39(Suppl 1): i48-i55. http://www.ncbi.nlm.nih.gov/pubmed/20348126.

Antimalarials - Artemesinin compounds for malaria

- **Definition:** Percent of children treated within 48 hours of the onset of fever in malaria-endemic areas with an artmesinin-containing compound (artemisinin-based combination therapy, or ACT).

- **Default data source:** Coverage data for this indicator are drawn from DHS, MICS, and other nationally representative household surveys. This includes Malaria Indicator Surveys.

- **Notes:** Historic data often measures treatment with any antimalarial in 48 hours, rather than ACTs specifically. These data can be used to create a historical trend, but there is not a good way to translate "any antimalarial" into an estimate of ACT coverage due to differing levels of effectiveness.

- **Effect size reference:** Thwing J, Eisele TP, Steketee RW. Protective efficacy of malaria case management for preventing malaria mortality in children: A systematic review for the Lives Saved Tool. BMC Public Health 2011; 11(Suppl 3): S14. http://www.ncbi.nlm.nih.gov/pubmed/21501431.

Therapeutic feeding for severe wasting (severe acute malnutrition (SAM))

- **Definition:** Percent of severely wasted children (<-3 Z-score) receiving therapeutic feeding. Therapeutic feeding is outpatient treatment including supplementation with ready-to-use therapeutic foods (RUTF) and maternal education.

- **Default data source:** Coverage data for this indicator are not typically available. Currently set at 0 for baseline; user should enter local data if possible and available.

- **Notes:** Therapeutic feeding is only applied to the percent of children severely wasted. It shifts children from the severely wasted category to moderately (-3 to -2 Z-score) and mildly (-2 to -1 Z-score) wasted categories.

- **Effect size reference:** Lenters LM, Wazny K, Webb P, et al. Treatment of severe and moderate acute malnutrition in low- and middle-income settings: A systematic review, meta-analysis and Delphi process. BMC Public Health 2013; 13(Suppl 3): S23. http://www.ncbi.nlm.nih.gov/pmc/articles/PMC3847503/.

  Bhutta ZA, Das JK, Rizvi A, et al. Evidence-based interventions for improvement of maternal and child nutrition: what can be done and at what cost? Lancet 2013; 382(9890): 352-77. http://www.ncbi.nlm.nih.gov/pubmed/23746776. (Supplemental material, page 15.)

Treatment for moderate acute malnutrition (MAM)

- **Definition:** Percent of moderately wasted children (-3 to -2 Z-score) receiving outpatient treatment including supplementation with ready-to-use supplementary foods (RUSF) and maternal education.

- **Default data source:** Coverage data for this indicator are not typically available. Currently set at 0 for baseline; user should enter local data if possible and available.

- **Notes:** Treatment for MAM shifts children from the moderately wasted category into the mildly wasted category (-2 to -1 Z-score).

- **Effect size reference:** Lenters LM, Wazny K, Webb P, et al. Treatment of severe and moderate acute malnutrition in low- and middle-income settings: A systematic review, meta-analysis and Delphi process. BMC Public Health 2013; 13(Suppl 3): S23. http://www.ncbi.nlm.nih.gov/pmc/articles/PMC3847503/.

  Bhutta ZA, Das JK, Rizvi A, et al. Evidence-based interventions for improvement of maternal and child nutrition: what can be done and at what cost? Lancet 2013; 382(9890): 352-77. http://www.ncbi.nlm.nih.gov/pubmed/23746776. (Supplemental material, page 15.)

Cotrimoxazole

- **Definition:** Coverage and effectiveness of AIDS and HIV interventions are specified in the AIM module.

- **Default data source:**

- **Notes:**

ART (for children)

- **Definition:** Coverage and effectiveness of AIDS and HIV interventions are specified in the AIM module.

- **Default data source:**

- **Notes:**

Wasting

When the "direct entry of wasting" option is checked in the LiST configuration menu, the wasting tab appears in the Coverage menu. Within this tab, users may modify wasting rates either through the "single indicator for wasting" option or the "detailed indicators for wasting" option.

The purpose of the single indicator is aggregate the bottom (worst) two wasting categories to create a single percentage of children who are wasted. (For example, if 2% of children are in the <-3 Z-score category and 5% of children are in the -3 to -2 z-score category, the single indicator will display "7% of children wasted.") It also aggregates across age groups, weighting by the number of months in each age range (i.e., <1 month is assumed to represent 1/60 of children, 1-5 months is assumed to represent 5/60 of children, etc.).

When the "single indicator" option is selected, the first (baseline) year of the single indicator will be grayed out and the rest of the years will be active. In the detailed display, the first year will be active (allowing users to edit baseline data here if necessary), but the following years will be grayed out. When the "detailed display" option is selected, the single indicator will remain visible but will be entirely grayed out, and the detailed display will be fully active.

Please note that in a healthy population (assumed to have a normal distribution of height for age), approximately 2.275% of the population will fall below -2 z-scores (i.e., more than 2 standard deviations

below the median). As a result, we do not recommend setting the single indicator for wasting at a rate below 2.275%, as this is below what is considered normal for a healthy population.

Wasting distributions

- **Definition:** Distribution of the percent of children falling into one of four Z-score categories for weight for height: <-3Z (severe wasting), -3 to -2Z (moderate wasting), -2 to -1Z (mild wasting), and >-1Z (not wasted). Wasting reflects acute undernutrition.
- **Default data source:** Data have been recalculated for consistency across countries, using DHS and MICS datasets. This also ensures that the values reflect the exact age groups of interest.
- **Notes:** Disaggregated data are not available for 0-1 and 1-5 months. As a proxy, the 0-6 month values are applied to both age groups.

Fertility risks

Maternal age and birth order

- **Definition:** Distribution of all births, categorized by the maternal age and the parity of the birth.
- **Default data source:** Data are drawn from DHS, MICS, and other nationally representative household surveys; the sum of all categories must add to 100%.
- **Notes:**

• **Effect size reference:** Kozuki N, Lee ACC, Silveira MF, et al. The associations of parity and maternal age with small-for-gestational-age, preterm, and neonatal and infant mortality: a meta-analysis. BMC Public Health 2013; 13(Suppl 3): S2. http://www.ncbi.nlm.nih.gov/pmc/articles/PMC3847520/. (Relative risks are from unpublished calculations associated with the paper.)

Birth intervals

- **Definition:** Distribution of all births, categorized by the number of months between births.
- **Default data source:** Data are drawn from DHS, MICS, and other nationally representative household surveys; the sum of all categories must add to 100%.
- **Notes:**
- **Effect size reference:** Kozuki N, Lee ACC, Silveira MF, et al. The associations of birth intervals with small-for-gestational-age, preterm, and neonatal and infant mortality: a meta-analysis. BMC Public Health 2013; 13(Suppl 3): S3. http://www.ncbi.nlm.nih.gov/pmc/articles/PMC3847557/. (Relative risks are from unpublished calculations associated with the paper.)
